# Actuation and design innovations in earthworm-inspired soft robots: A review

**DOI:** 10.3389/fbioe.2023.1088105

**Published:** 2023-02-21

**Authors:** Jianbin Liu, Pengcheng Li, Siyang Zuo

**Affiliations:** Key Laboratory of Mechanism Theory and Equipment Design of Ministry of Education, Tianjin University, Tianjin, China

**Keywords:** soft robotics, earthworm-inspired robot, bioinspired systems, peristaltic locomotion, actuation method innovation, design innovation

## Abstract

Currently, soft robotics technologies are creating the means of robotic abilities and are required for the development of biomimetic robotics. In recent years, earthworm-inspired soft robot has garnered increasing attention as a major branch of bionic robots. The major studies on earthworm-inspired soft robots focuses on the deformation of the earthworm body segment. Consequently, various actuation methods have been proposed to conduct the expansion and contraction of the robot’s segments for locomotion simulation. This review article aims to act as a reference guide for researchers interested in the field of earthworm-inspired soft robot, and to present the current state of research, summarize current design innovations, compare the advantages and disadvantages of different actuation methods with the purpose of inspiring future innovative orientations for researchers. Herein, earthworm-inspired soft robots are classified into single- and multi-segment types, and the characteristics of various actuation methods are introduced and compared according to the number of matching segments. Moreover, various promising application instances of the different actuation methods are detailed along with their main features. Finally, motion performances of the robots are compared by two normalized metrics-speed compared by body length and speed compared by body diameter, and future developments in this research direction are presented.

## 1 Introduction

A robot is a machine, especially one that can be programmed to automatically perform a complex series of actions (Latombe, 2012). In general, robots can be controlled and guided by external or built-in control devices. Most robots today are machines that are used to perform tasks, designed with an emphasis on practicality and functionality. Beyond all doubt, robots have replaced humans in performing repetitive and dangerous tasks which humans prefer not to do, or are unable to do because of size limitations, or which take place in extreme environments (Akins, 2013). Many task-performing robots are used in the production lines of factories engaged in manufacturing industry (Kumar, 2013). Other application areas include injection molding industry, construction industry, oil drilling, ore mining, space exploration, underwater exploration, toxic material cleaning, search and rescue, medicine, military and other fields. At present, there are two main types of robots, based on their use: general-purpose autonomous robots and dedicated robots. Except for this, robots can be subdivided according to the specificity of their purpose. At present, various technologies have emerged to develop robotics and robot science (Alimisis, 2013). Many future applications of robotics seem obvious, although they far exceed the capabilities of existing robots as predicted (Dahiya and Valle, 2013). In order to solve practical issues of mechanical technology, or to develop new machines and technologies, human beings begin to gain experience through observation of nature and create a new discipline–bionics (Tavsan and Sonmez, 2015). With the advent of bionics, robots based on the movement principles of actual plants and animals began to receive widespread attention. Bionic robots such as folded-wing robots (Calvente et al., 2021), robot birds (Couceiro et al., 2010), and miniature robotic insects (Li et al., 2012) have been developed and have created a new idea for research and development of this type of robot and solved many practical problems.

Rigid robotics is a mature field that has been extensively reviewed, so this review does not include relevant content. On another front, soft robotics is a subfield of robotics that concerns the design, control, and fabrication of robots composed of compliant materials, instead of pure rigid links (Rus and Tolley, 2015). It should be noted that “soft” does not specifically imply that the entire robot is completely composed of soft materials. Rather, even in the presence of rigid components, it is still possible to reflect the flexible or continuously changing characteristics of the components during movement. Compared to traditional rigid robots, a soft robot is more suitable for bionic movements with higher operational stability and safety (Rus and Tolley, 2015). Soft robots offer enormous potential for deformation as they may be shaped by external pressures to advance in intricate constrained places (Hawkes et al., 2017). In fact, their soft structure allows them to bend or squeeze and reduces stress caused by contact between the surrounding environment and the surface (Coevoet et al., 2019). These features have sufficient advantages for some special fields. However, there are still some urgent problems that need to be solved for soft robots. For example, soft robots are still difficult to control as precisely as rigid robots. Existing sensing and control components are still mostly rigid and difficult to integrate effectively with soft robots, which inevitably increases the overall stiffness (Whitesides, 2018).

On some specific issues, for example, in order to solve the problems of pipeline inspection and gastrointestinal tract inspection, humans have drawn inspiration from nature and finally found that the above problems could be solved efficiently by imitating the movement of earthworms. In recent years, earthworm-inspired soft robots have garnered tremendous attention owing to its peristaltic locomotion characteristics with motion stability and adaptability for executing unstructured motion scenarios. Nowadays, earthworm-inspired soft robots have been extensively developed, with key application scenarios involving in-pipe inspection (Consumi et al., 2022), soil drilling (Dewapura et al., 2020), medical endoscopy (Wang et al., 2009), surface crawling (Yu et al., 2022), etc.

The earthworm movement process is relatively uncomplicated. Therefore, earthworm-inspired soft robots have made great achievements in recent years as related research has intensified. In general, researchers mainly use earthworm-inspired soft robots for two purposes, one for the examination of the human gastrointestinal tract and the other for the inspection in pipelines. There are now a number of robots that have been put into practical use. With the deepening of the research on the principle of earthworm locomotion and the inspiration of interdisciplinary research, this kind of robot has also begun to expand to other fields of application. Although these robots come in different shapes and actuation methods, some issues are the same. First, most robots do not resemble the actual movement of earthworms, and some even move forward by their own vibration. Second, many of the designs mentioned in the article lack a controllable tip to install cameras or associated detection components. Some current designs have placed rigid components such as flanges inside the robot segment, a practice that undoubtedly increases the overall stiffness. The structure, purposes and related issues of the earthworm-inspired soft robot will be described and discussed in the subsequent chapters.

**FIGURE 1:**
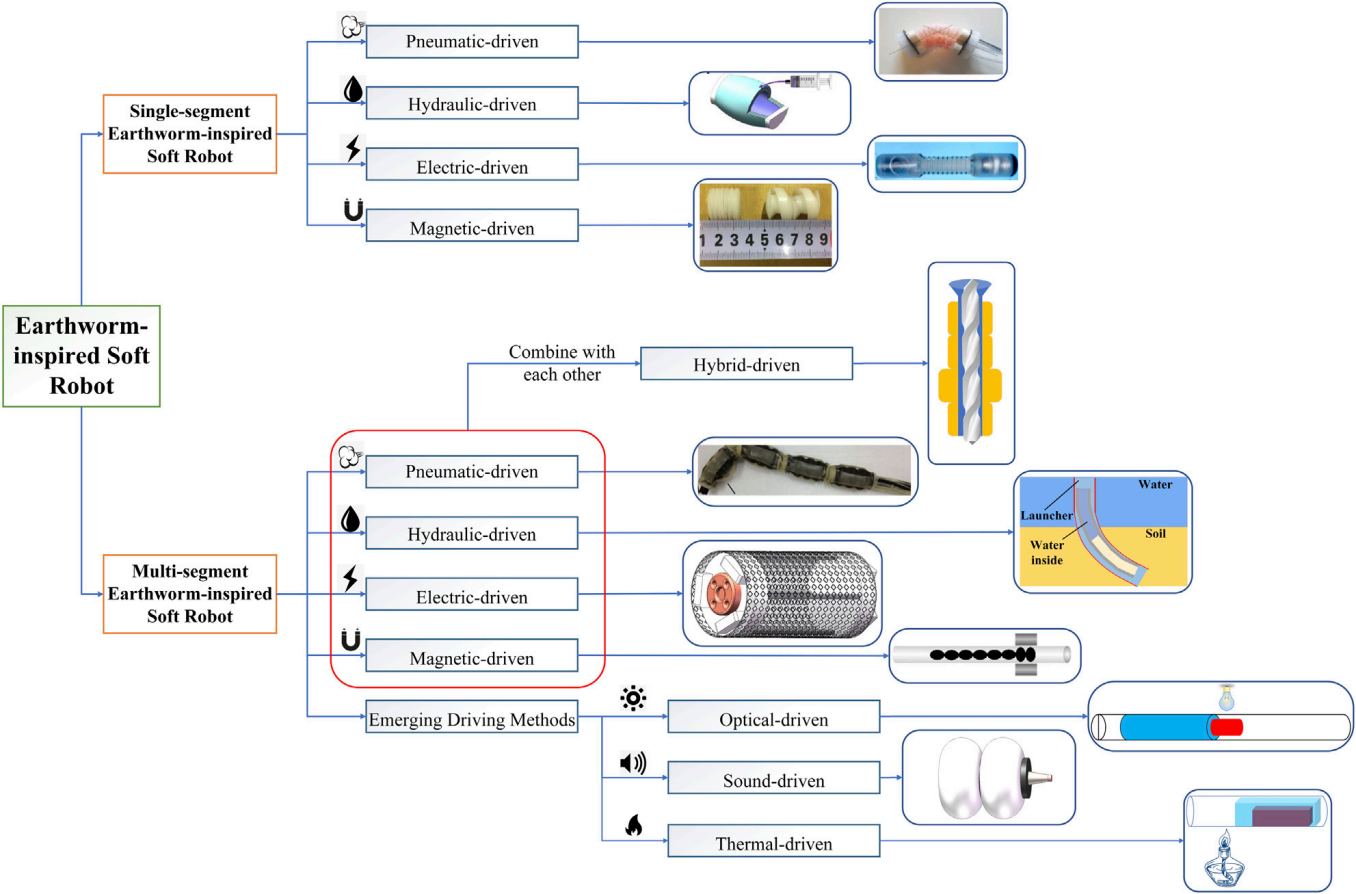
Chart of various types of earthworm-inspired soft robots discussed in this review article, classified based on actuation methods and number of body segments (Kim et al., 2006; Yanagida et al., 2012; Wang et al., 2018a; Guo et al., 2018; Hu and Cheneler, 2021; You et al., 2021). From Ref. (Guo et al., 2018; Hu and Cheneler, 2021; You et al., 2021), used under Creative Commons CC-BY license. Reproduced from Ref (Kim et al., 2006; Yanagida et al., 2012; Wang et al., 2018a) with permission.

Currently, there are several reviews that mention earthworm-inspired soft robots in their descriptions in the field of engineering application (Dario and Mosse, 2003; Kheirikhah et al., 2011; Kim et al., 2013; Gu et al., 2016; Wei et al., 2021; Dong et al., 2022) and robotic gastroscopes (Marlicz et al., 2020; Manfredi, 2021). But detailed classification, actuation analysis and discussion of such robots are still lacking. In this Review, the principles of earthworm locomotion and related biological characteristics have been introduced, and the advantages of earthworm-inspired robot locomotion are described. Overall, the current state-of-the-art models of earthworm-inspired soft robots can be further classified into two major categories according to their locomotion form: single-segment and multi-segment earthworm-inspired soft robots. Within each category, they are further subdivided based on their various actuation methods, as illustrated in Figure 1. Moreover, this paper details the locomotion features, actuation methods, and the relative merits and demerits of such robots. Among them, the suitability of various actuation methods is discussed and explained through specific examples and their structural optimization process is described. Ultimately, we explore the future development potential of the various categories of earthworm-inspired soft robots.

With the optimization of actuation methods, earthworm-inspired soft robots can be expanded beyond a single form of pressure-driven. Other actuation methods such as magnetic-driven and electric-driven, have achieved significant breakthroughs, and the combination of pressure-driven and other actuation methods produces a more efficient form of the hybrid-driven method. Furthermore, emerging actuation methods, such as sound-driven, optical-driven and thermal-driven, have been demonstrated as well. These new contactless actuation methods are promoting the future development of earthworm-inspired soft robots.

Therefore, certain earthworm-inspired soft robots with distinctive features and great potential are selected for this review. This review mainly focuses on innovations in robot structures or actuation methods/form of energy conversion, while the improvement of control system is not involved. Kinematic performance of the robot is also analyzed and compared by two normalized metrics - velocity corresponding to body length and velocity corresponding to diameter, respectively. In the subsequent references cited in this review, the motion speed of the robot is described using the absolute velocity divided by the body length (bl·s^–1^). Finally, we analyze the actuation method with the best motion performance and provide insights into the future development of earthworm-inspired soft robots. The basis of classification and drafting mentality of this Review article are illustrated in Figure 1.

Here, the biological characteristics of earthworms will be discussed first, and their locomotion advantages will be elaborated. Frontier applications based on other directions of earthworm biological characteristics will also be briefly presented.

## 2 Biological characteristics of earthworms and related robots on this foundation

### 2.1 Biological characteristics of earthworms.

An earthworm is a terrestrial invertebrate in the phylum Annelida (Ansari and Ismail, 2012). The fundamental biological characteristics of an earthworm’s segments are bilateral symmetry with external segmentation and corresponding internal segmentation (Edwards and Bohlen, 1996). Additionally, it contains a double transport system comprising coelomic fluid that moves within the fluid-filled coelom and a simple-but-efficient circulatory system (Patel and Prajapati, 2020). In particular, earthworms use dielectric mechanisms to stay clean as they move through moist surroundings. Due to this mechanism, an electrical charge is generated on the outer surface of the cell wall, which creates a layer of immobile ions that allows the earthworm to move smoothly in the soil (Wandall et al., 2022). As invertebrates, earthworms do not bear any skeleton, but they maintain their structure through fluid-filled coelom cavities that function as a hydrostatic skeleton (Gutmann, 1981). Therefore, the hydrostatic skeleton structure has garnered extensive attention owing to the incompressibility of liquids and the dearth of rigid structures. Other than that, Earthworms have the ability to regenerate lost segments, but this ability varies between species and depends on the degree of damage.

**FIGURE 2:**
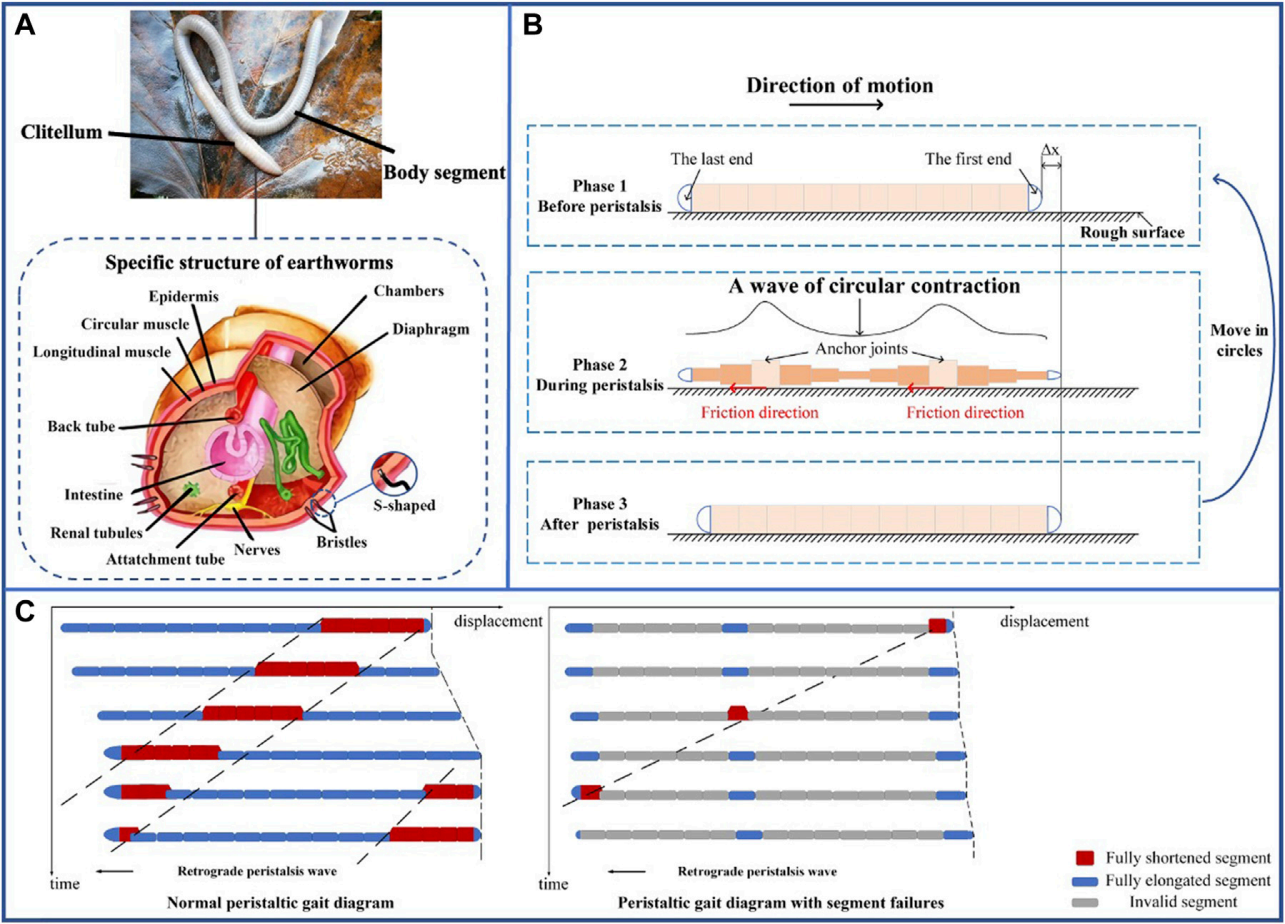
Structure and motion of earthworms. **(A)** Specific structure of earthworms. **(B)** Schematic of earthworm movement and motion variation diagram of earthworm body segments. **(C)** Peristaltic gait diagram with or without segment failures.

Earthworms wriggle underground by expanding and contracting its various segments, and a wave of circular contraction passes backward over its body (Gray and Lissmann, 1938). During burrowing, the internal pressures of the earthworm body segments increase to a much higher level (Gray and Lissmann, 1938; Newell, 1950). In addition to the anchored body segments, the earthworm pushes the front end of itself forward into the soil by pushing the remaining body segments. Thereafter, other joints then continue to move forward as the new anchor joints (Menciassi et al., 2004a). Although the earthworm bears a relatively simple structure, it is extremely efficient in its concertina movement owing to the hydrostatic skeleton noted earlier. According to anatomical findings, the body segments of the earthworm are composed of circular and longitudinal muscles (Tzetlin and Filippova, 2005). As depicted in Figures 2A, B, if the segment diameter is shortened, the longitudinal muscles stretch and the circular muscles contract accordingly to ensure volume conservation of the body segments. Correspondingly, if the segment diameter is elongated, the longitudinal muscles shorten and the circular muscles stretch. Moreover, friction occurs between the contracted body segments and ground, and a reaction force enables them to extend and reciprocate in such a manner. Ultimately, the earthworm can continue to move forward. The movement of earthworms is not only deformation of its own segments, but also the wagging of its body as shown in Figure 2A, which enables it to find an easier path ahead. This also leads to two ideas for subsequent research on earthworm-inspired robots - mimicking the deformation of earthworm segments and mimicking the wagging of the earthworm.

Depending on the species, an adult earthworm can be from 10 mm (0.39 in) long and 1 mm (0.039 in) wide to 3 m (9.8 ft) long and over 25 mm (0.98 in) wide, but the typical Lumbricus terrestris grows to about 360 mm (14 in) long. And the longest earthworm on confirmed records is Amynthas mekongianus, which can reach up to 3 m (10 ft) in length and up to 8 mm (0.3 in) in width. Crawling speed varies both within and among individuals. The crawling speed of earthworms on wet ground is about 5 mm·s^–1^ (Gu et al., 2016), and the drier the place, the slower the crawling speed. Generally, the ratio of the static diameter/length of earthworm segment is greater than 1, rendering each segment a discus-like shape. In a single body segment, because the volume of this segment is conserved, the muscles of earthworms can contract in the direction of diameter during their movement changes, resulting in larger length changes (Quillin, 1998). To improve the peristaltic efficiency, earthworms contain a ring of S-shaped bristles surrounding the periphery, except the first two segments to improve peristaltic efficiency (Plisko and Nxele, 2015; Ortiz et al., 2019). Moreover, the longitudinal muscles of the earthworms produce a greater force (Hidaka et al., 1969) to creep through the cracks in the soil layer. Prior experiments have reported on the forces measured according to body weight: larval earthworms can push 500 times of their own body weight, whereas adult earthworms can only push 10 times of their own body weight (Quillin, 2000).

With the rapid development in the field of bionics, researchers are devoted to the study of earthworm-inspired soft robots for motion control, actuation methods, bionic motion and other related aspects.

### 2.2 Advantages of earthworm movement and related robots on this foundation.

After analyzing the actual motion of the earthworms, researchers apply the peristaltic movements of the earthworms to the motion of the soft robots. The movement process of earthworms has been described in detail earlier. The advantages of the earthworm-inspired soft robot can be elaborated as follows.

1. Inspired by the continuous peristaltic movement of earthworms, such robots can achieve multimodal movement, that is, it can use the same form of locomotion to travel through long pipelines (Consumi et al., 2022), mud (Dewapura et al., 2020) or on surface (Yu et al., 2022). Although there are some robots that advance by vibration (Tang et al., 2022), such robots can produce a certain degree of expansion and contraction in segment deformable designs. In this review, the maximum relative axis deformation of single segment is up to approximately 950% (from 86 (+43) mm to 8.2 (+43) mm, where 43 mm is the space between adjacent segment) of its own length (Fang et al., 2017a). Whereas the maximum relative radial deformation of single segment is up to 275% of its own diameter (from 20 mm to 75 mm) (Chen et al., 2022). The deformation produced in the radial direction is, in general, infinitely adjustable and highly adaptable for pipes of different diameters (Consumi et al., 2022).
2. For untethered robots, they can continue to move as long as the power source and structures remain active, while the range of movement of cabled robots is determined by the length of wireline. The robot can be bent and stretched through a special structure and regular telescopic gait.
3. This kind of robot is fault-tolerant and reliable, especially for multi-segment earthworm-inspired soft robots. Specifically, when a structural failure is encountered, as shown in Figure 2C, if the number of segments of the robot is greater than X (X ≥ 3), only X–3 segments fail, but the robot is still able to move forward with lower efficiency.

Soft robots inspired by earthworm motion are the main content described in this review. Application examples and corresponding structural optimization will be described in subsequent chapters.

### 2.3 Robots based on other biological characteristics of earthworms

Based on other biological characteristics of earthworms, there are a number of cutting-edge ideas in addition to the earthworm-inspired soft robots mainly described in this review.

**FIGURE 3:**
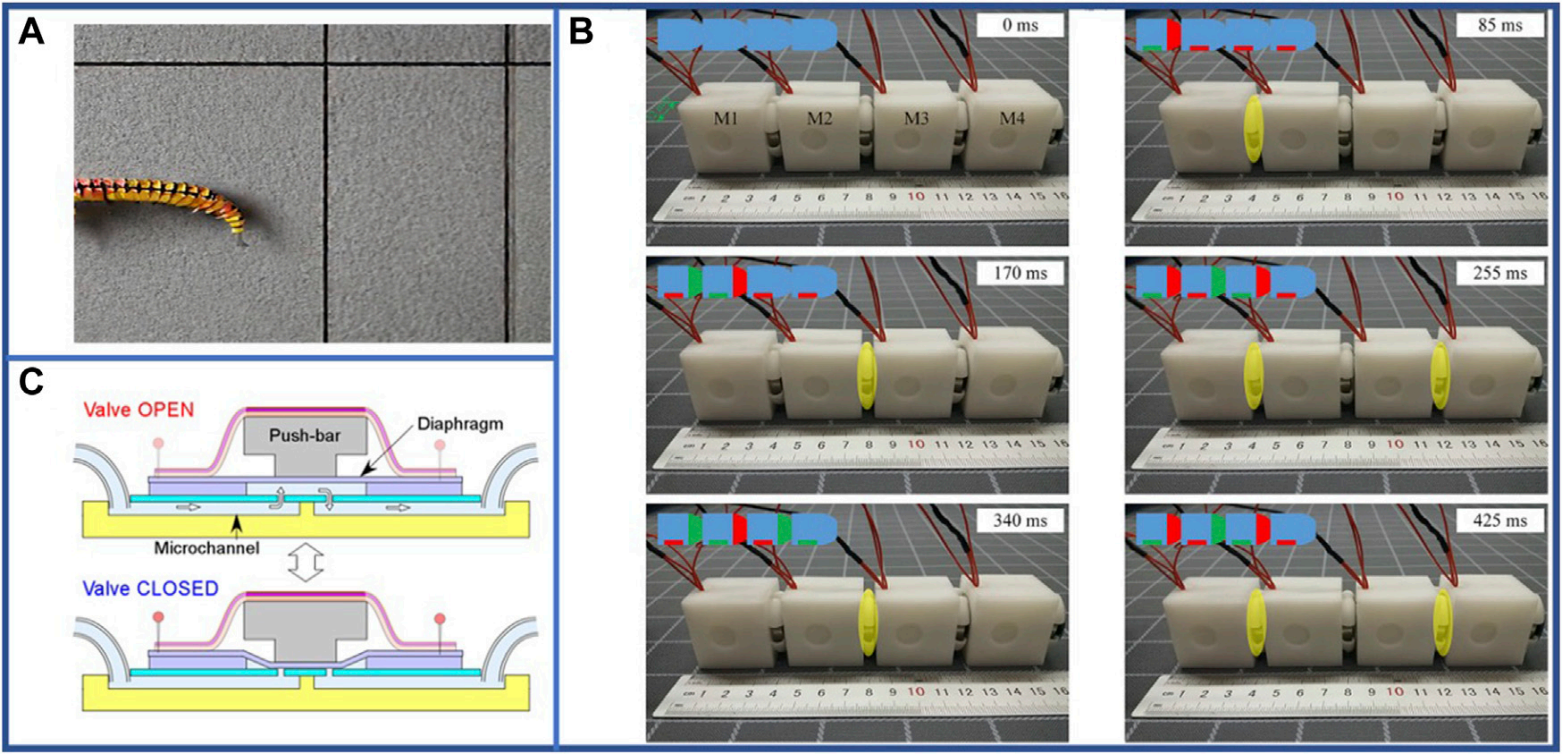
Some cutting-edge ideas based on earthworms. **(A)** Earthworm-inspired wall crawling robot attached to vertical walls. **(B)** Modular wall crawling robot using electromagnetic effect and its movement process (Zhang et al., 2021a). From Ref. (Zhang et al., 2021a), used under Creative Commons CC-BY license. **(C)** A valve powered by earthworm muscle with chemical control (Tanaka et al., 2019). From Ref. (Tanaka et al., 2019), used under Creative Commons CC-BY license.

Wall crawling is an innovation based on the earthworm’s peristaltic process. The most important feature of wall-climbing robots is that it can significantly improve the adsorption capacity of the bristle-like structure. Earthworms in nature are unable to crawl on vertical walls, and researchers have used piezoelectric motors (Zhou et al., 2013), suckers (Yang et al., 2018) or electromagnetic effects (Zhang et al., 2021a) attach the robot to vertical walls and perform earthworm-like crawling, as shown in Figure 3A. This design allows the robot to climb vertical walls of even walls with obtuse angles, an ability that is currently lacking in conventional earthworm-inspired soft robots. Modular designs have also emerged for rapid replacement of damaged body segments, as shown in Figure 3B (Zhang et al., 2021a). Although the speed of the robot is slightly slower, it can climb the vertical wall with stability. Its load capacity and reliability are also generally stronger than traditional climbing methods.

With the in-depth study of actual earthworm skin, some research results of self-healing artificial skin based on earthworm have been published. Earthworms are attached with periodic folded skin and exhibit excellent robustness to cohesive soils (Xu et al., 2021). Therefore, this excellent adaptability was mimicked and used on superhydrophobic (SH) fabrics, which were treated in this way to provide extraordinary superhydrophobic durability (Carmichael, 2021; Ruan et al., 2022). In addition, SH fabrics are self-healing after heat or plasma treatment. This approach paves a new path for designing ultra-durable and fluorine-free fabrics. It also demonstrates the possibility of achieving ultra-durable superhydrophobic fabrics by creating soft, adaptable skins with surface morphology and gradient modulus.

In addition to this, the development of bio-microactuators that combine microdevices and cellular mechanical functions has been an active area of research owing to their desirable properties including high mechanical integrity and biocompatibility. The control of muscle contraction and diastole by chemical (acetylcholine) stimulation allows the observation of prolonged (more than 2 min) and reproducible displacement. The force generated by the muscle can control the valve opening and closing, just as shown in Figure 3C, and the pressure resistance can reach 1.5 kPa (Tanaka et al., 2019).

## 3 Single-segment earthworm-inspired soft robots

Single-segment earthworm-inspired soft robot implies that the robot’s overall structure can be deemed as a single segment of the actual earthworm, which can produce axial and radial expansion even if there may be multiple actuators. Owing to the limited volume of a single segment, these robots typically employ a single actuation method, e.g., pneumatic-driven, hydraulic-driven, electric-driven, or magnetic-driven, instead of multiple driven. Although there may be multiple actuators, current single-segment robots usually have no more than three active actuators in total, and the internal systems of such robots are much simpler and less difficult to manufacture and control. As a consequence, this kind of robot has the advantages of relatively simple structure and convenient control. However, it bears the disadvantages of poor fault tolerance and substandard biomimicry owing to discontinuous stepping motion.

### 3.1 Pneumatic-driven

In the evolution of the single-segment earthworm-inspired soft robot, the first application of actuation methods involved pneumatic-driven robots owing to their controllability, economy, and reliability. The robot is primarily composed of silicone, soft rubber, and other soft materials, and it is simulated by inflation and deflation for expansion and contraction. Previously, the robot relied predominantly on the scalability of the material itself to imitate earthworms (Qi et al., 2015). Non-etheless, with improvements in valve design, air path optimization and additional manufacturing methods such as 3D printing (Lin et al., 2022), robots are able to easily construct more complex structures and can achieve superior performance.

Generally, the movement of pneumatic-driven robots in a pipeline simulates that of a circular muscle through a part of longitudinal expansion and contraction. Additionally, it then simulates the movement of longitudinal muscle through another part of axial expansion and contraction.

**FIGURE 4:**
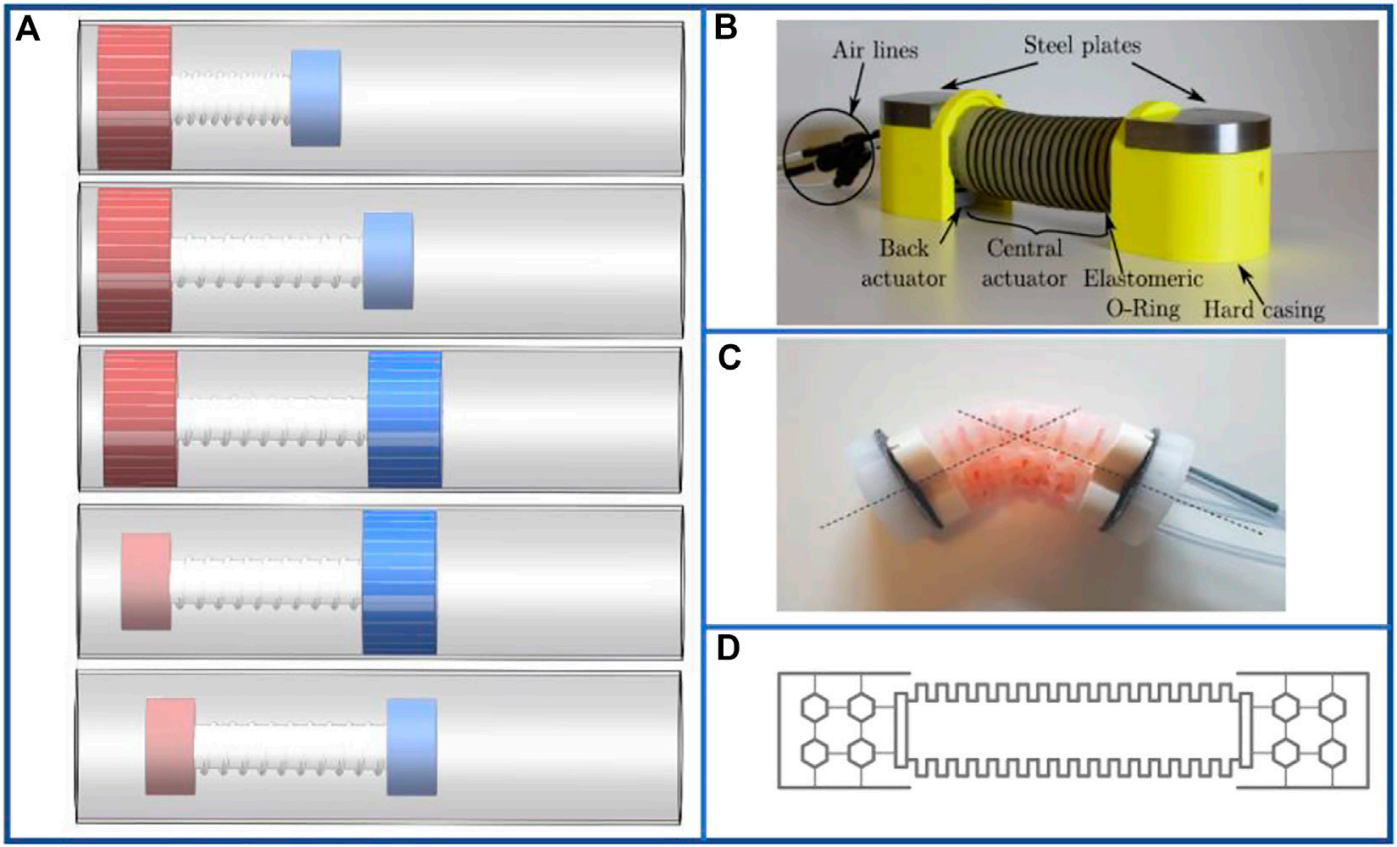
Examples of various structures of pneumatic-driven single-segment earthworm-inspired soft robot. **(A)** Fiber-covered pneumatic-driven robot schematic and order of actuation for peristaltic motion. **(B)** Configuration of surface crawling pneumatic-driven robot (Joey et al., 2017). Reproduced from Ref. (Joey et al., 2017). with permission. **(C)** Bending diagram of pneumatic-driven robot for pipe inspection with VAMP structure (Hu and Cheneler, 2021). Reproduced from Ref. (Hu and Cheneler, 2021). with permission. **(D)** Structural diagram of pneumatic-driven robot with cellular structure.

Pneumatic-driven robots travelling in the GI tract of humans majorly use an extensor-based actuator—completely composed of soft materials—to enhance security and reduce material hardness (Phee et al., 2002; Manfredi et al., 2019; Pan et al., 2022) which has attained a locomotion speed of 0.0014 bl·s^–1^ (Phee et al., 2002). Furthermore, the soft actuator adapts to the curvature of the intestinal or gastric tract, enabling the entire robot to move faster. Moreover, materials with higher softness are selected to significantly increase the efficiency of movement in biological pipes such as the gastric tract. The most commonly used structure for single-segment pneumatic-driven robots is based on the Soft Pneumatic Inchworm Double balloon (SPID) (Manfredi et al., 2019). This structure consists of three main pneumatic actuators, in the order of the anchoring-telescopic-anchoring parts, as shown in Figure 4A. The above three actuators act in sequence to accomplish a movement similar to that of one body segment of real earthworms. Compared to materials of the moving segments, those of fixed anchor segments need to be softer and have larger deformation (Manfredi et al., 2019; Pan et al., 2022). Accordingly, a steerable pneumatic-driven robot suitable for gastrointestinal examinations has been developed to adapt to the folds, depressions, and curves of the biological intestine, creating smoother movements and posing less impact on the organism (Heung et al., 2016; Manfredi et al., 2019; Pan et al., 2022). Presently, the robot designed by Heung et al. using steering structure is currently moving at a speed of 0.015 bl·s^–1^ under the pressure of 75 kPa (Heung et al., 2016).

In contrast, linear actuators with better telescopic performance are more common for pneumatic-driven robots adapting to abiotic environments (Joey et al., 2017; Li-ming et al., 2018; Xavier et al., 2019a; Xavier et al., 2019b; Calderón et al., 2019; Dewapura et al., 2020; Hayashi et al., 2020) such as in-pipe locomotion and surface crawling. The research on pneumatic-driven robots crawling in abiotic pipes initially focused on the elasticity of the material itself (Li-ming et al., 2018; Dewapura et al., 2020). The speed of the robot designed by Dewapura et al. is 0.06 bl·s^–1^ with a pressure of 20 kPa for all actuators (Dewapura et al., 2020). To improve material elasticity, the linear actuator has been encapsulated by fibers (Xavier et al., 2019a; Xavier et al., 2019b) or O-rings (Calderón et al., 2019), which can significantly improve the locomotion speed and stability. To increase the movement distance of a single inflation, bellows (Chen et al., 2022) have been used to increase the elongation distance during inflation. The separation of air input control for the fixed anchor segment and linear actuator of the robot can achieve improved locomotion efficiency. In particular, in wet condition of the complex pipe, as the friction factor between the outer surface of the robot and the inner wall of the pipe becomes smaller, slippage is more likely to occur if the same control method as in the dry case is still used. Thus, the contact area (Hayashi et al., 2020) or the coefficient of friction (Lin et al., 2022) must be increased by varying the actuator material. With such a structure, the robot can smoothly pass through a complex pipe with a locomotion speed of about 0.058 bl·s^–1^ without stuck (Hayashi et al., 2020). Although the surface-crawling pneumatic-driven robot—imitating the earthworm bristles—is fixed at one end with increased friction between the anchor and ground, it can travel forward by expansion of the linear actuator as shown in Figure 4B. In this case, the robot designed by Joey et al. can travel at an average speed of 0.1 bl·s^–1^ under the pressure of 1.2 kPa, 3 kPa for rear/front actuator and middle actuator, respectively (Joey et al., 2017).

With the progressive development of applied cross-disciplinary and precision machining methods, the structures of pneumatic-driven robots are in focus of optimization. In context, the Origami structure, vacuum-actuated muscle-inspired pneumatic (VAMP) structure (shown in Figure 4C), cellular structure (shown in Figure 4D), and other new structures have been widely used in the linear actuators of the earthworm-inspired soft robot owing to their high flexibility and stability. In the Origami structure, the single movement displacement distance of the pneumatic-driven robot is significantly increased and the force retention is improved (Jiang and Pei, 2021). Although the linear ductility of the VAMP structure is less than that of the Origami structure, it delivers improved bending performance and can realize the controllable bending movement of the earthworm-inspired soft robot (Hu and Cheneler, 2021). Furthermore, it can convert one-dimensional motion into two-dimensional or even three-dimensional steering peristalsis. The cellular structure integrates a single pneumatic actuator and achieves the elongation and anchoring motion of the earthworm (Liu et al., 2020) that considerably simplifies the motion control problems.

Self-healing capability can be also achieved by incorporating a self-healing silicone elastomer (PDMS-TFB) into a conventional silicone material (Jiang et al., 2020). Mixed materials can be heated to accelerate the rate of self-healing of the material, and performance of the robot is almost completely restored after healing. This approach greatly improves the service life of the soft robot.

### 3.2 Hydraulic-driven

As earthworms themselves are filled with tissue fluid, hydraulic-driven robots deliver the same driving principle as the actual earthworm, thereby yielding better biological similarity. However, most hydraulic-driven soft robots are still in their infancy because of issues such as volume limitations and challenges in manufacturing materials under high operation pressures, which forms the scope for further improvement. Because the hydrostatic skeleton is responsible for the flexible movement of earthworms in the soil, the application of hydrostatic skeletons is currently the focus of research on hydraulic-driven robots.

**FIGURE 5:**
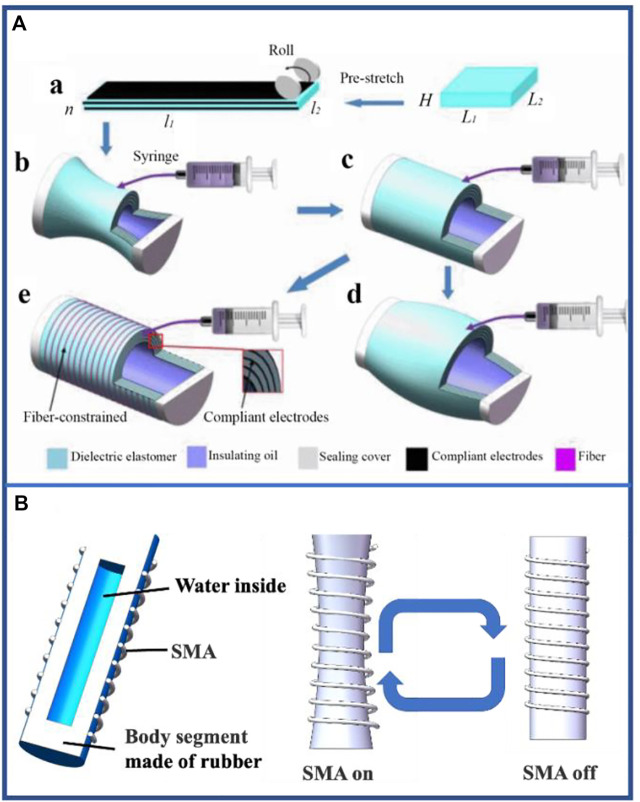
Basic principle and examples of hydraulic-driven single-segment earthworm-inspired soft robot. **(A)** Conceptual fabrication process and structural expansion during liquid injection. Two pieces of a DE membrane with the same designed pre-stretch ratio are stacked with three coats of compliant electrode alternatively and then rolled into a DE tube with two caps (a). With the injection of insulating oil, the DE tube will go through three stages: shrinking stage (b), cylinder stage (c) and bulging stage (d). However, when the cylinder DE tube is constrained in the circumferential direction, the DE tube will maintain a cylinder shape and elongate to the design goal (e). Reproduced from Ref. Wang et al. (2018a) with permission. **(B)** Operation diagram of SMA actuating hydraulic structure.

Owing to the incompressibility of liquids, visible deformations occur to a certain extent when liquids are injected into or out of enclosed cavities composed of soft materials as shown in Figure 5A (Wang et al., 2018a). Evidently, the inflow and outflow of liquid create the following issues for single-segment hydraulic-driven robots: liquid density is generally large, and the mass of single-segment of the robots is much larger than that of pneumatic-driven robots; hydraulic-driven control requires high manufacturing technology and high cost. However, this is precisely because of the incompressible feature of liquids and shape memory alloys (SMA) can scale by switching on and off that it can be used as a new method to drive materials for scaling without the inflow and outflow of liquids as shown in Figure 5B (Matsushita et al., 2022), which considerably varies the stability of motion and increases the amount of deformation of materials. The control enacted by SMA eliminated the structural components of the pump and valve required in the traditional hydraulic-driven method, and it avoided the challenges such as liquid leakage, which provides a greater reference value for the subsequent development of hydraulic-driven earthworm-inspired soft robots.

### 3.3 Electric-driven

**FIGURE 6:**
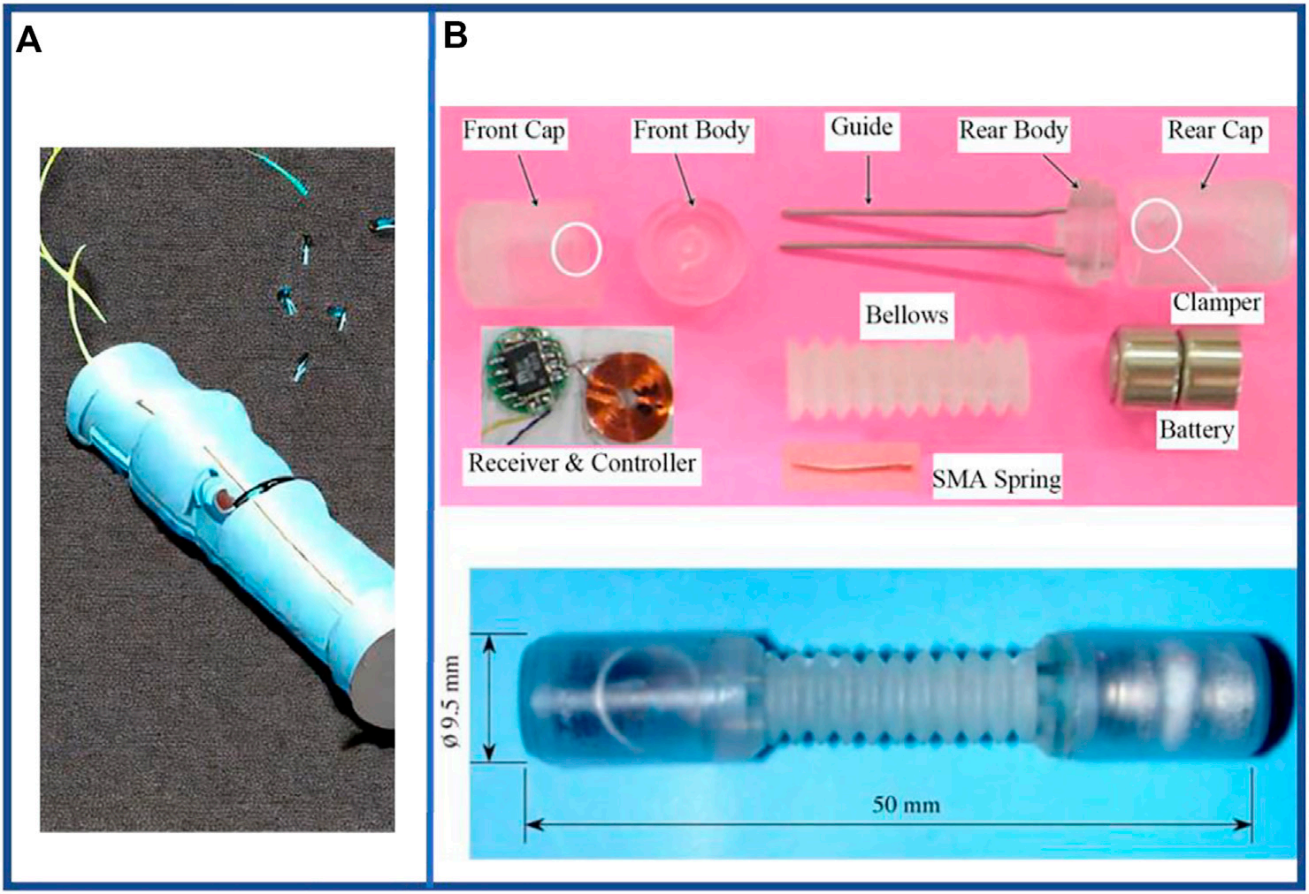
Examples of electric-driven single-segment earthworm-inspired soft robot. **(A)** Mini electric-driven earthworm-like robot used for capsule endoscopes. **(B)** Drawings of components and assembly of microrobot using shape memory alloy actuator (Kim et al., 2006). Reproduced from Ref. (Kim et al., 2006). with permission.

Generally, the form of the electric-driven facilitates the compactness of the robot structure. In context, the electric-driven method provides certain advantages in terms of variety of miniaturized electric linear drives and rotary drives. Moreover, it exhibits no sealing problems, and thus, offers much better controllability in comparison to pneumatic- and hydraulic-driven methods. The configuration of a typical electric-driven robot is shown in Figure 6A.

Currently, the motor-driven wire retraction enables a single-segment electric-driven robot to achieve a bending function (Yu et al., 2022) that is similar to that of an actual earthworm. This control method enables the robot to adjust the original unidirectional displacement into a controlled angle of bending, in addition to moving forward *via* its own twisting motion. Yu et al. (2022) have designed a robot based on this structure that can move at a speed of approximately 0.0675 bl·s^–1^. In addition, the electric-driven method enables robots to drive untethered (Kim et al., 2005) along with a considerably reduced volume occupation and improved integration of components as shown in Figure 6B.

Furthermore, robot can incorporate an SMA to compress the material and enforce the actuator structure for exerting external forces that generates the axial elongation and longitudinal compression of the earthworm-inspired motion. In principle, SMA is an alloy that can be deformed when cold but reverts to its pre-deformed (“remembered”) shape when heated. The shape memory effect (Qader et al., 2019) of SMA can be used to drive robots. Therefore, SMA can manipulate the electric-driven earthworm-inspired robot with power supply, i.e., the SMA contracts when energized, causing the robot to compress axially; when the power is cut off, the SMA resumes elongation, enabling the robot to elongate axially. The microrobot designed by Kim et al. (Kim et al., 2006) can move at a speed of 0.0075 bl·s^–1^. Specifically, it needs to be powered on for 2 s and powered off for 6 s to complete the cooling work during movement. This reflects the disadvantages of the slow response and sensitivity of SMA to external temperature.

Owing to the advantages of high integration, simple structure, convenient control and adequate stability, the electric-driven earthworm-inspired soft robot is more inclined to multi-segment structure, which is discussed in the following sections.

### 3.4 Magnetic-driven

**FIGURE 7:**
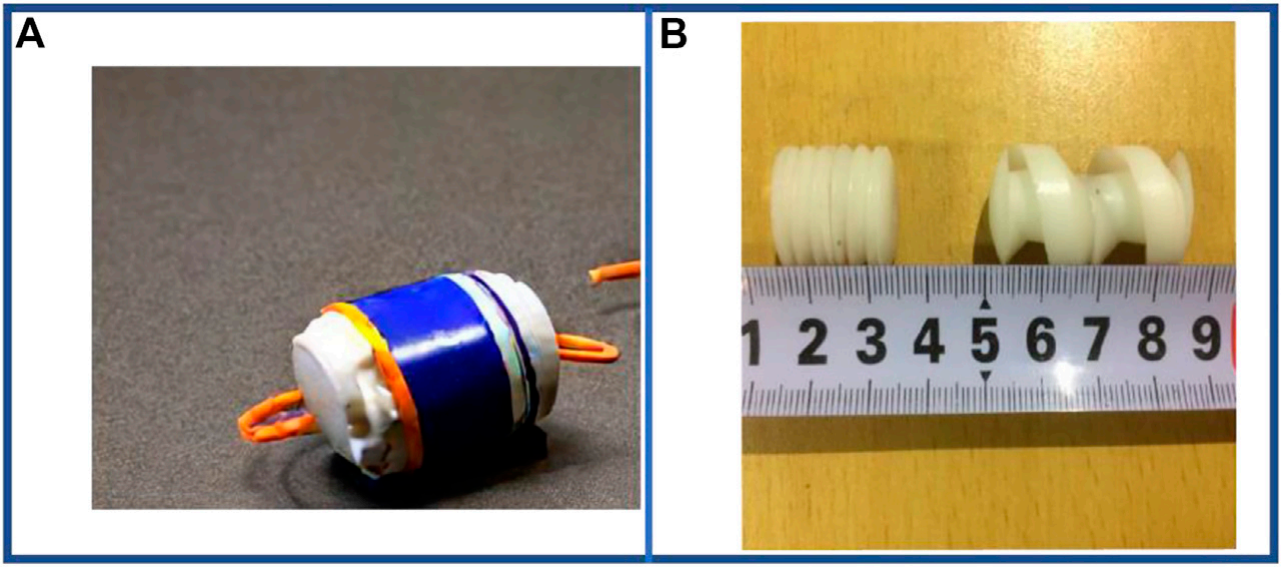
Examples of magnetic-driven single-segment earthworm-inspired soft robot. **(A)** Magnetic-driven capsule robot prototype. **(B)** Comparison of deformation of body segments (Guo et al., 2018). From Ref. (Guo et al., 2018), used under Creative Commons CC-BY license.

Magnetic-driven is a technology that transmits force or torque (power) by applying a magnetic force that is generated by a permanent magnet or an external magnetic field (Zhang et al., 2021b). The configuration of a typical magnetic-driven robot is shown in Figure 7A.

Generally, the magnetic-driven method can be classified into two major actuation forms, i.e., using the internal magnetic pole rotation of the actuator to produce axial expansion and contraction movement, or by varying the external magnetic field to produce deformation in the exterior surface layer of the actuator, containing the magnetic powder, to enable displacement. Regardless of applying any of the two forms stated above, the untethered motion can be facilely achieved and the motion can be realized without a built-in power source. Therefore, this actuation method provides high safety. However, as the magnetic field strength is inversely proportional to the square of the distance, the efficiency of the movement is reduced over long distances.

External magnetic field is mainly used as the power source of the single segment robot. Due to the small size of such robot, and in order to cope with the different diameters of the gastrointestinal tract between different individuals, it is generally necessary to design variable-diameter units as shown in Figure 7B. With the spiral leg deformation (Zhang et al., 2010; Chen et al., 2014), even small-sized robots can move over a relatively wide range of diameters. Most of these robots are driven by an external coil with a rectifier circuit that generates electricity from a ferrite core inside the segment.

Due to the difficulty of material preparation and the anchor problem of single-segment robot, the vast majority of researchers are using magnetic-driven more often in multi-segment types, as discussed in the subsequent chapters.

## 4 Multi-segment earthworm-inspired soft robots

Multi-segment earthworm-inspired soft robots refer to the robot’s overall structure can be viewed as more than one segment or is similar to the entire body of an actual earthworm, which can produce axial and radial expansion as a whole. Generally, such robots adopt pneumatic-driven, hydraulic-driven, electric-driven, magnetic-driven, or a combination of the above actuation forms (hybrid-driven) for locomotion. With the development of materials engineering, emerging actuation methods such as optical-driven, sound-driven and thermal-driven have been developed as well. Currently, the multi-segment earthworm-inspired soft robot constitutes the main trend of the research on earthworm-inspired robots as it is relatively more efficient and has better fault-tolerance.

### 4.1 Pneumatic-driven

**FIGURE 8:**
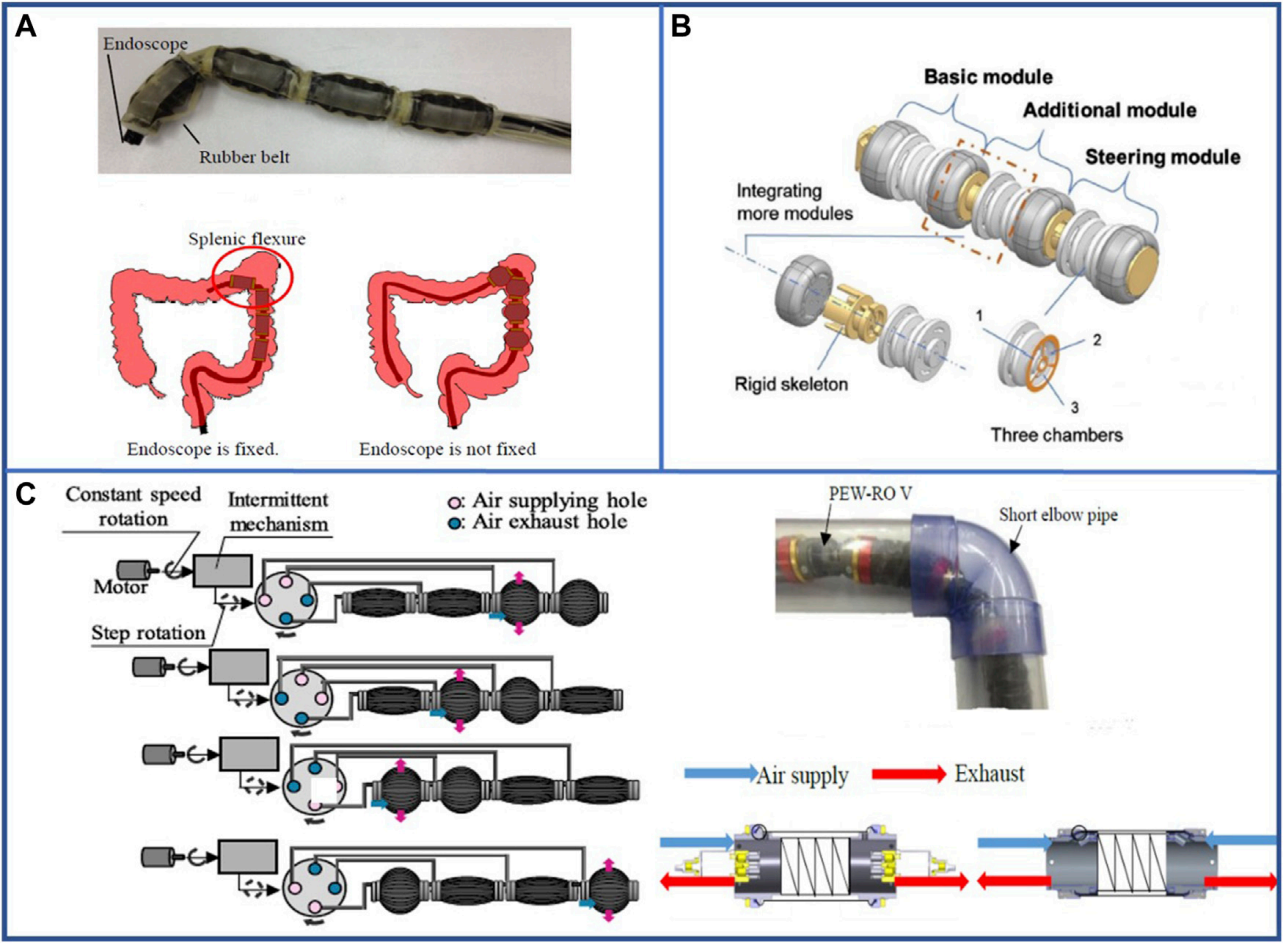
Examples of pneumatic-driven multi-segment earthworm-inspired soft robot for pipeline inspection. **(A)** Bending diagram of segment and experimental methodology (Yanagida et al., 2012). Reproduced from Ref. (Yanagida et al., 2012). with permission. **(B)** Concept design and fabrication of soft robot with multiple actuators connected in series and demonstrations of steering module bending to random directions in 3D space (Liu et al., 2022). Reproduced from Ref. (Liu et al., 2022). with permission. **(C)** Appearance of robot locomotion and the new pneumatic valve (Sato et al., 2021). Reproduced from Ref. (Sato et al., 2021). with permission.

The multi-segment pneumatic-driven robots have been improved accordingly in order to allow smoother operation of the gastrointestinal tract. Early multi-segment pneumatic-driven robots were rigid endoscopes overlaid with an inflatable silicone membrane, along with an inflatable catheter inside. By inflating the catheter, the silicone membrane could be inflated, and an earthworm-like peristaltic simulation was accomplished by the expansion and contraction of the material itself (Slatkin et al., 1995). The inclusion of a rigid structure, on the other hand, resulted in poor biocomfortability, and the subsequent development of a pneumatic-driven robot suitable for gastroscopy with a purely soft material effectively improved this problem (Hoeg et al., 2000). At the same time, robots that can bend and anchor have been developed (Yanagida et al., 2012) to cope with the bends and folds of the biological gastrointestinal tract. In addition, the robot in Figure 8A uses bellows inside (Hoeg et al., 2000; Yanagida et al., 2012) to significantly increase the forward distance of a single movement, so that its maximum locomotion speed reaches 0.009 bl/s (Yanagida et al., 2012). In order to reduce the control difficulty and the amount of input ports, the segments can be connected in series actuating in sequence controlled by small orifices that delay the flow between segments (Glozman et al., 2010). This way of relying on the fluid flow dynamics between inflatable segments connected in series requires only one air input line for the deformation of the multi-segment structure.

Pipeline inspection is a crucial application area of pneumatic-driven multi-segment robots, and certain robots have already entered the practical application stage offering appreciable social and economic benefits. Upon separating the air chamber of a single segment into multiple chambers, the robot can be steered by inflating a specific air chamber (Liu et al., 2022), as shown in Figure 8B, enabling the robot to automatically select a path and complete a pipeline inspection through machine learning. The speed of the 500 mm soft robot designed by Liu et al. (2022) in a horizontal, tilted, and vertical pipe was 0.0101, 0.00916, and 0.00846 bl·s^–1^, respectively, when the pressure was 85 kPa for the foot actuators and 60 kPa for the body actuators. The majority of the pneumatic-driven robots used for pipeline inspection are equipped with springs (Ito et al., 2021) or bellows (Ono and Kato, 2010; Ishikawa et al., 2016; Ishikawa et al., 2017; Tomita et al., 2017) inside the segment section or with special expansion units (Kamata et al., 2017; Kamata et al., 2018) to significantly increase the expansion performance. For instance, the robot developed by Ono and Kato, 2010 internally used bellows to achieve a locomotion speed of 0.103 bl·s^–1^ at the moving distance of 30 m. And the air pressure of the robot (Ono and Kato, 2010) is set at + 10 kPa for positive pressure and −40 kPa for negative pressure in the three bellows. With the modification of the control valve as shown in Figure 8C (Sato et al., 2021), two segments can be inflated in a single cycle that doubles the distance of movement per cycle. Thus, the locomotion speed can be doubled as well. For the pneumatic-driven robots used for pipeline inspection, the rigid component of the robot has been increased to improve the structural and movement stability. Moreover, the steering inside the pipeline can be accomplished by adding a torsion unit (Ikeuchi et al., 2012; Mano et al., 2018a; Mano et al., 2018b; Uchiyama et al., 2021). These pneumatic-driven robots have been practically used to achieve operating speeds over 0.01525 bl·s^–1^ at an applied pressure of 0.05 MPa (Mano et al., 2018b), and they can be operated in wet pipelines as well (Uchiyama et al., 2021).

**FIGURE 9:**
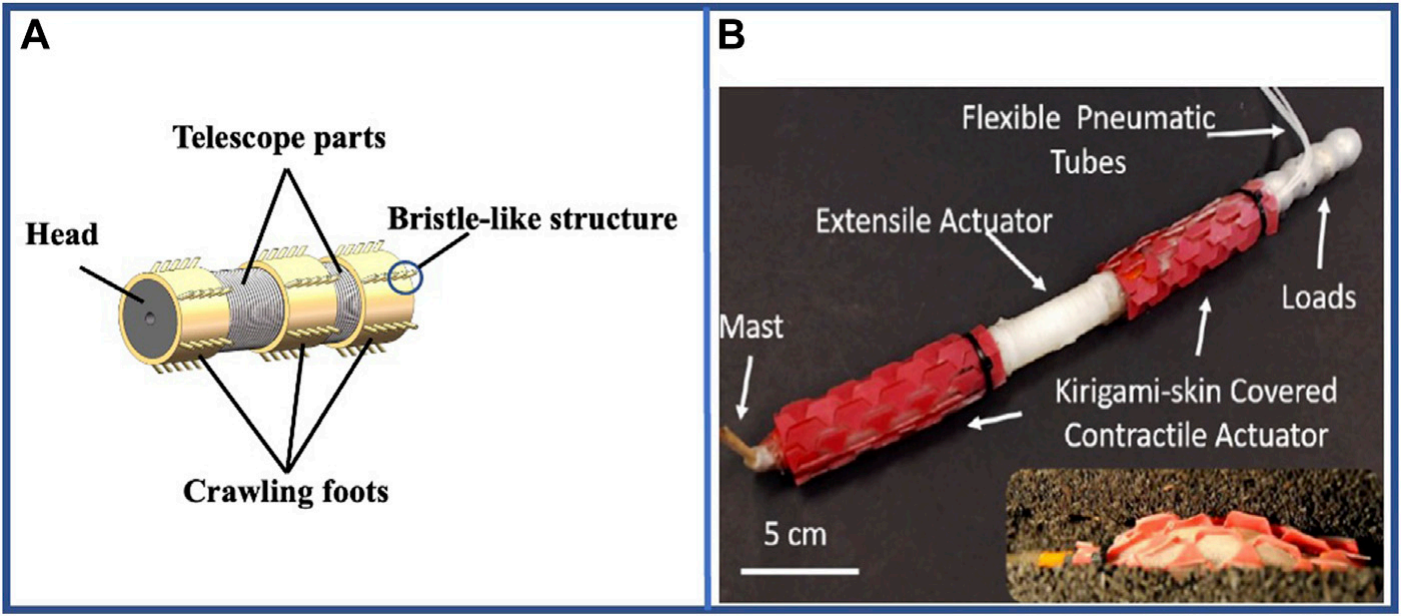
Examples of pneumatic-driven multi-segment earthworm-inspired soft robot for surface crawling and related new structures and applications. **(A)** Model of the typical soft robot for surface crawling. **(B)** Earthworm-inspired soft robot with Kirigami skin (Liu et al., 2019). Reproduced from Ref. (Liu et al., 2019). with permission.

To use pneumatic-driven robots used for surface crawling, a structure that imitates earthworm bristles is usually added to their outer surface. A typical robot structure for surface crawling is shown in Figure 9A. This method increases the coefficient of friction on the outer surface of the robot and effectively prevents slippage. Zhou et al. have designed a robot with a pneumatic telescopic unit and bristle-like structure, just as shown in Figure 9A, to crawl forward about 35 mm after a period of motion (Zhou et al., 2016). Similarly, steerable crawling can be achieved on the surface by performing air chamber separation on a single body section (Aydin et al., 2018; Goldoni et al., 2020; Ozkan-Aydin et al., 2021). Consequently, certain fascinating new designs have been proposed. For instance, a single segment can be individually designed by bonding low- and high-hardness materials together to form a complete segment that realizes various states of expansion and contraction, which can achieve controllable bending (Tang et al., 2020). This robot can be applied to a complex environment such as unstructured pipeline owing to its flexible shape and appropriate self-adaption, and the 3-segment robot can travel at speeds of up to approximately 0.0078 bl·s^–1^ under the pressure ranging from 13.8 kPa to 15.2 kPa.

Multi-segment pneumatic-driven robots can also be used in other fields: with airbag disposal, the robot can be used to transport objects through its inner channel, and the objects are squeezed by sequentially inflated airbags to realize the transportation (Nakamura, 2022).

To fulfill the requirements of the above-stated applications, new structures such as Kirigami and Origami are used on multi-segment pneumatic-driven robots. The Kirigami structure adheres to the surface of the pneumatic actuator to provide a relatively larger anchor force in the mud by popping out instantaneously with the radial expansion of the actuator. This motion creates bristle-like spikes perpendicular to the surface, which folds downward when deflated to create a smoother skin structure, just as shown in Figure 9B (Liu et al., 2019). Pneumatic-driven robot using Origami structure combines the structural advantages derived from the morphological characteristics of earthworms and the benefits from Origami folding. The Origami structure facilitates significant expansion and contraction of the individual segments, which enables them to attain a displacement of approximately 40 mm (Fang et al., 2017b). In addition, the structure can also be twisted to facilitate turning of the robot (Li et al., 2021; Ze et al., 2022). For instance, Ze et al. designed a surface-crawling robot using Origami structure can complete the O-shaped movement in 60 s (Ze et al., 2022).

### 4.2 Hydraulic-driven

Hydraulic-driven multi-segment robots have always been one of the challenging research areas for earthworm-inspired soft robots. The current limitations include engineering materials that are capable of carrying denser liquids, controlling the flow of liquids, and determining the material parameters that cannot be easily evaluated.

**FIGURE 10:**
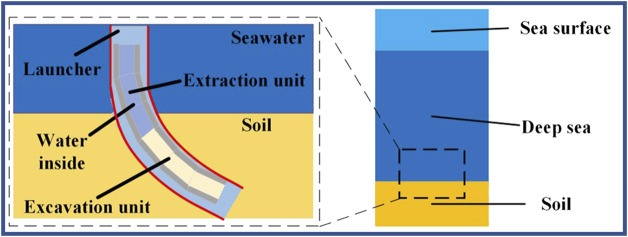
Model of robot operation in seafloor.

Presently, certain hydraulic robots using purely rigid materials (Fang et al., 2018) exhibit stable motion and appropriate load-bearing capacity, but they still pose the issue of large structure size and weight. Currently, most hydraulic robots are employed in operation under high-pressure environment such as underwater (Nagai et al., 2015). The basic structure and the position of the underwater movement are basically similar to those depicted in Figure 10. Thus, the actuators need to meet the requirements of water-proof, high-pressure resistance, and high output force, which cannot be achieved by ordinary pneumatic-driven robots. Overall, hydraulic-driven multi-segment earthworm-inspired soft robots are still in the early stage of research and require further investigation.

### 4.3 Electric-driven

Generally, electric-driven multi-segment robots offer size reduction, convenient control. And superior integrity. Electric-riven robots can be classified into two main categories: generalized motor-driven and electro-deformable material-driven. Furthermore, generalized motor-driven can be subdivided into direct motor-driven, wherein the motor impels the wire to drive the robot (hereinafter, referred to as wire-driven), and the motor drives other continuous deformable structures. Among the electro-deformable material-driven multi-segment robots, the SMA-driven and dielectric elastomer (DE)-driven are the two methods most commonly used. All these five methods exhibit its own characteristics, which will be discussed and analyzed later. In particular, the new continuous deformable structure enables continuous and flexible structural changes similar to that of an actual earthworm.

**FIGURE 11:**
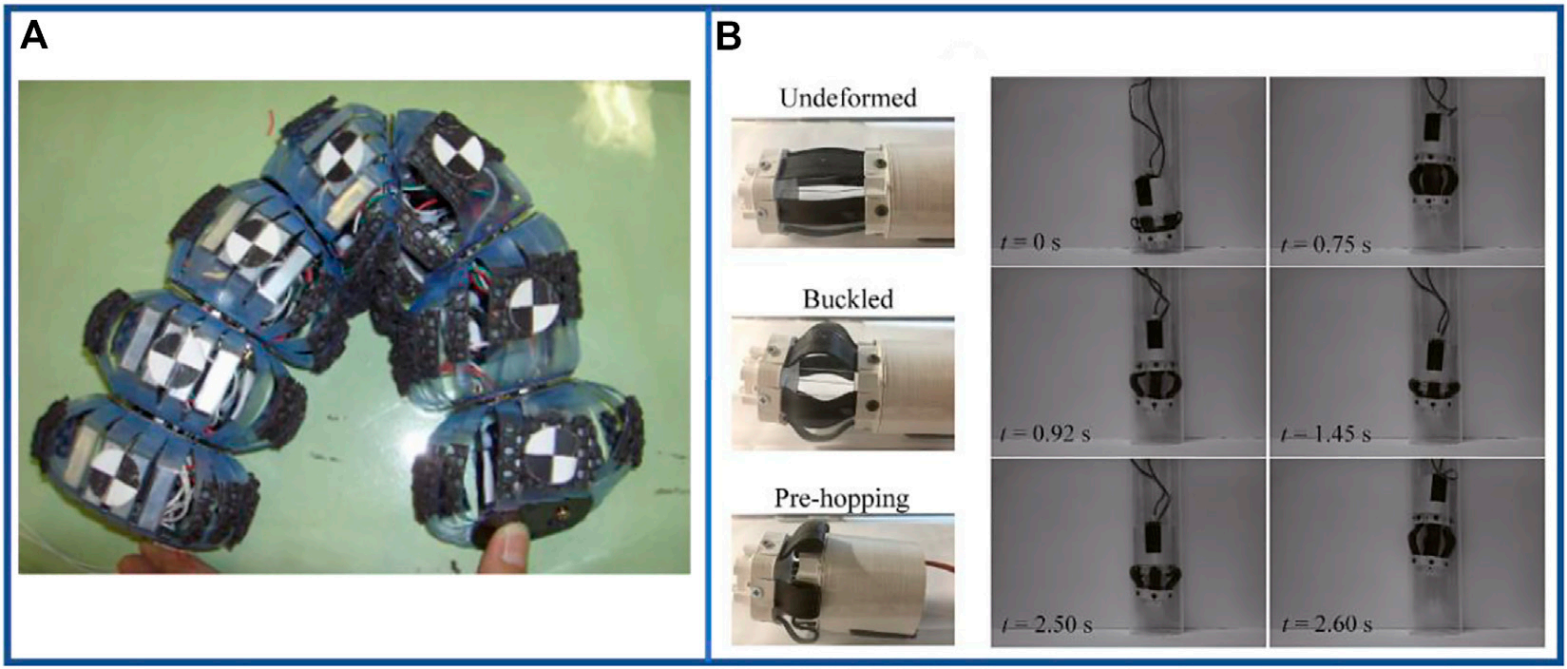
Examples of direct motor-driven multi-segment earthworm-inspired soft robots. **(A)** Diagram of angle control and bending of robot segment (Omori et al., 2008). Reproduced from Ref. (Omori et al., 2008). with permission. **(B)** Three states of single-section robot and representative images illustrate vertical hopping process of single-section robot in continuous hopping (Yeh et al., 2020). Reproduced from Ref. (Yeh et al., 2020). with permission.

Direct motor-driven is a simple and efficient actuation method that employs rotary or linear motor to drive each segment for self-operation for achieving forward motion. At all instances, direct motor-driven multi-segment robots exhibit a faster running speed and can be more easily used in practical scenarios. The direct motor-driven robot (Zuo et al., 2005) can elongate a body segment and accomplish continuous forward motion by driving the subsequent body segments. To achieve faster movement, the additional elastic parts such as springs and bellows (Wang et al., 2017) can be used to increase the telescopic length. Wang et al. (2017) proposed a miniaturized motor-driven gastrointestinal tract inspection robot that was able to travel at a speed of about 0.021 bl·s^–1^ in experiment. Similarly, certain new structures can deliver adequate stretching and simulation performance: the linkage mechanism can realize bending function, which can significantly stretch the single segment and can also attach the bristle-like structure to the linkage mechanism for realizing the contraction function (Takahashi et al., 1995). In particular, the scissor structure can perfectly combine the axial shortening and longitudinal elongation together (Niu et al., 2015) to complete the simulation of longitudinal and circular muscles with a single structure. To alter the linear forward motion (Nakamura et al., 2006) into a controllable angle steering motion (Nakamura and Iwanaga, 2008; Omori et al., 2008), the linear servo motor can be replaced with an angular servo motor as shown in Figure 11A. Subsequently, a large axial deformation can be achieved by the torsion of the motor that drives the elastic belt to expand. Yeh et al. (2020) designed a robot that can hop inside the pipeline and attain a speed of 0.0177 bl·s^–1^ when crawling in a horizontal pipe as shown in Figure 11B. The inherent passivity of low-torque DC motors in each linear joint can be used as a control law (Masuda et al., 2018) to drive robots, which demonstrates that autonomous and distributed creep wave generation can be achieved without using sensors, controllers or microprocessors. In addition, the earthworm mimetic manipulator architecture (EMMA) mechanism (Kannapan et al., 2021) can realize the lockable and force-effective motion capability that is adjusted according to the earthworm gait and provides a new concept for future steering design. Thus, new structures and control components can be incorporated into direct motor-driven robots to enhance the telescoping performance of single segments. As discussed earlier, Origami structures (Fang et al., 2017a; Fang et al., 2017b) offers precision, low-cost manufacturing, and high degree of customizability with greater scalability and lower control difficulty. The 724 mm long robot developed by Fang et al. (2017a) can attain a locomotion speed of 0.0215 bl·s^–1^ if both the segments remain in an axially shortened state (i.e., radially expanded) and anchored with the environment.

**FIGURE 12:**
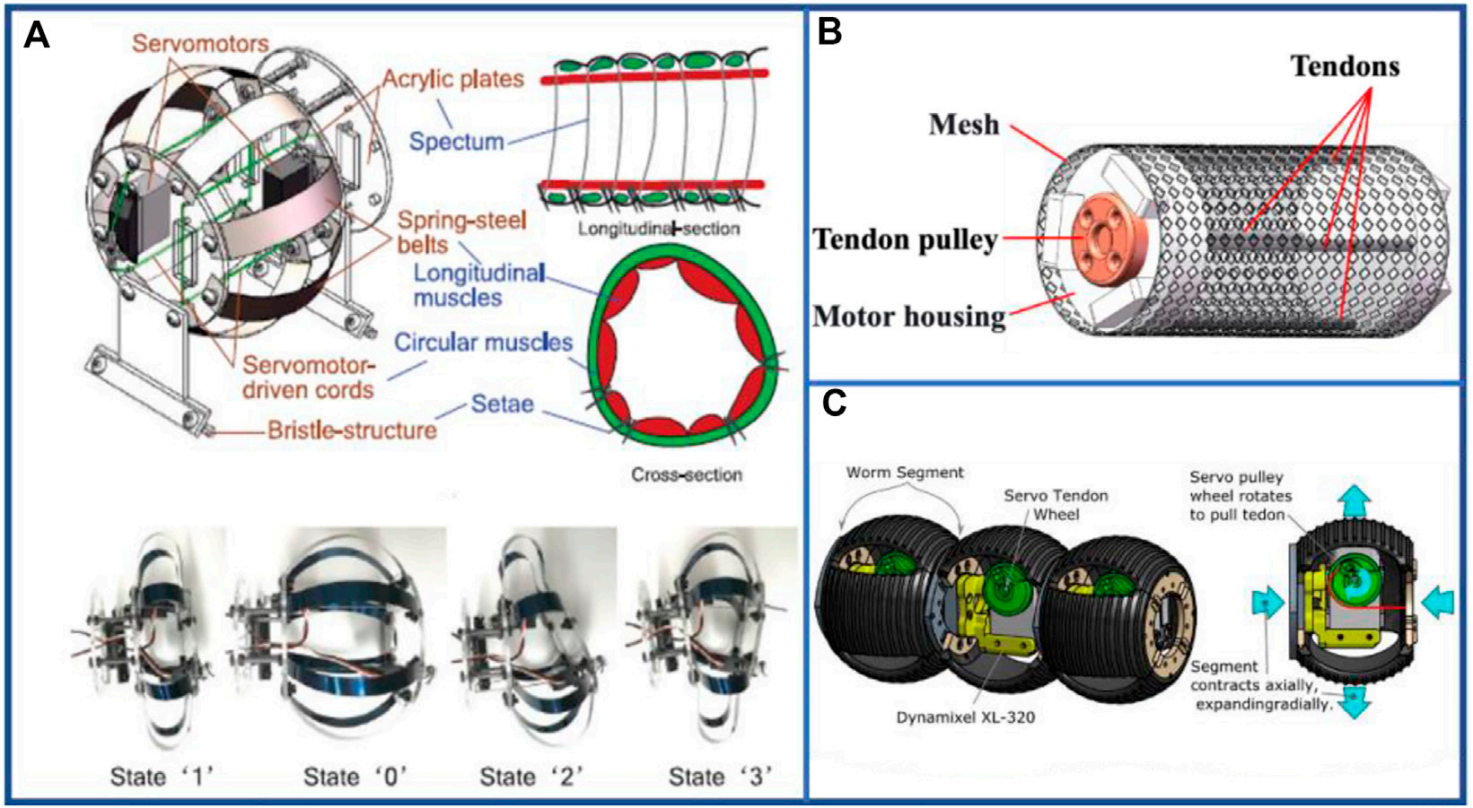
Examples of wire-driven multi-segment earthworm-inspired soft robots. **(A)** Design and prototype of earthworm-like locomotion robot (Zhan et al., 2019). Reproduced from Ref. (Zhan et al., 2019). with permission. **(B)** 3D model of segment of typical wire-driven robot. **(C)** Dynamixel driven robot constructed to test each waveform and compare performance and identify optimal gearbox parameters (Winstone et al., 2016). Reproduced from Ref. (Winstone et al., 2016). with permission.

The wire-driven method provides a convenient approach to achieve continuous actuation and locomotion of the robot, in which the stretch distance and rotation angle size can be infinitely controlled by the retracting length of the wire. With the addition of a wire controller on the exterior of the segment, the effective wire length in the segment can be reduced by twisting (Fang et al., 2014; Zhan et al., 2019) (shown in Figure 12A) or traction (Manwell et al., 2014), thereby causing simultaneous contraction and expansion in the axial and longitudinal directions of the segment, respectively. After completing the action, it can be reset to the initial state. And the basic configuration of a common typical wire-driven robot is shown in Figure 12B. The robot fabricated by Thomas et al. (Manwell et al., 2014) has been able to attain locomotion speeds of up to 0.0175 bl·s^–1^ on a flat surface and the braided mesh and spring achieved the earthworm-like motion, creating an efficient and safer alternative for human endoscopy. Simultaneously, certain groups of wires can be used to achieve the steering function. In particular, steering in a specific direction can be achieved by controlling the movement of a specific group of wires (Zhan et al., 2019). The wire can be wound in the segment by the worm movement as shown in Figure 12C (Winstone et al., 2016), which can alter the direction of force transmission and effectively reduce volume occupation.

**FIGURE 13:**
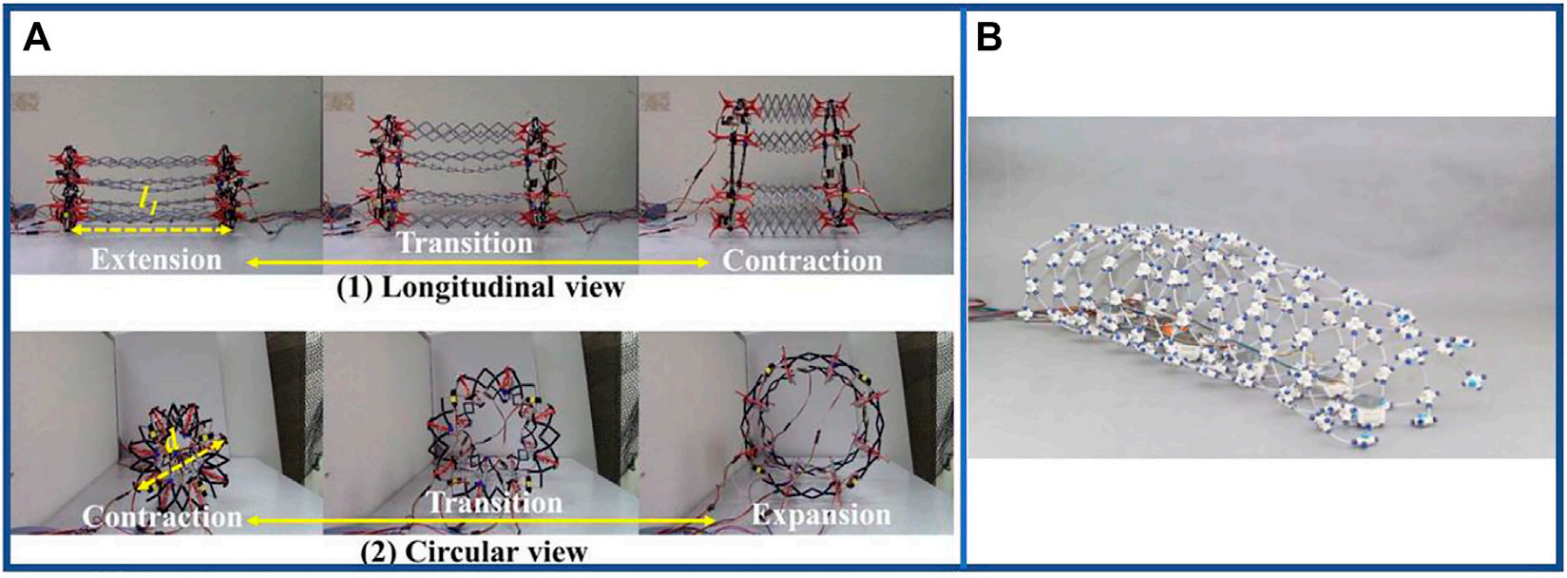
Examples of electric-driven multi-segment earthworm-inspired soft robots with continuous deformable structures. **(A)** Design and deformation of earthworm-like locomotion robot (Luo et al., 2022). Reproduced from Ref. (Luo et al., 2022). with permission. **(B)** Prototype of worm robot controlled by central pattern generator (Wang et al., 2018b). Reproduced from Ref. (Wang et al., 2018b). with permission.

To achieve a continuous and consistent variation, various continuous deformable structures have been proposed for earthworm-inspired robots. Currently, these robots constitute an entire rigid body, but if they are covered with a layer of soft material, they can achieve an overall continuous movement and have a shape similar to that of an actual earthworm. The scissor mechanism (Luo et al., 2016) and morphing mechanism (shown in Figure 13A) (Luo et al., 2022) driven by motor can simulate the circular and longitudinal muscles to enact continuous and consistent movement. A class of rigid elements-based morphing structures employs various 2D scissor elements to achieve 3D deformation. For instance, Luo et al. designed a robot using morphing structures (Luo et al., 2022) that delivered a competitive locomotion speed both in absolute speed (122.4 mm s^–1^) and body lengths per minute (4.67–9.42). The outer mesh (Boxerbaum et al., 2011; Daltorio et al., 2013; Wang et al., 2018b) and its derivatives (Horchler et al., 2015) proposed by Horchler et al. provided an evolution in the simulation of earthworm. Instead of containing discrete body segments, this structure makes the robot a whole, and facilitates continuous and consistent actuation. Moreover, this structure exhibits wave-shaped variations (Zhou et al., 2016) that are extremely similar to real earthworms. The robot shown in Figure 13B designed by Wang et al. (Wang et al., 2018b) can achieve a speed of 0.01 bl·s^–1^ on flat pavements.

Unlike the generalized motor-driven, the electro-deformable material-driven eliminates the involvement of rigid motors. Overall, it provides a new idea for further development of electric-driven robots.

**FIGURE 14:**
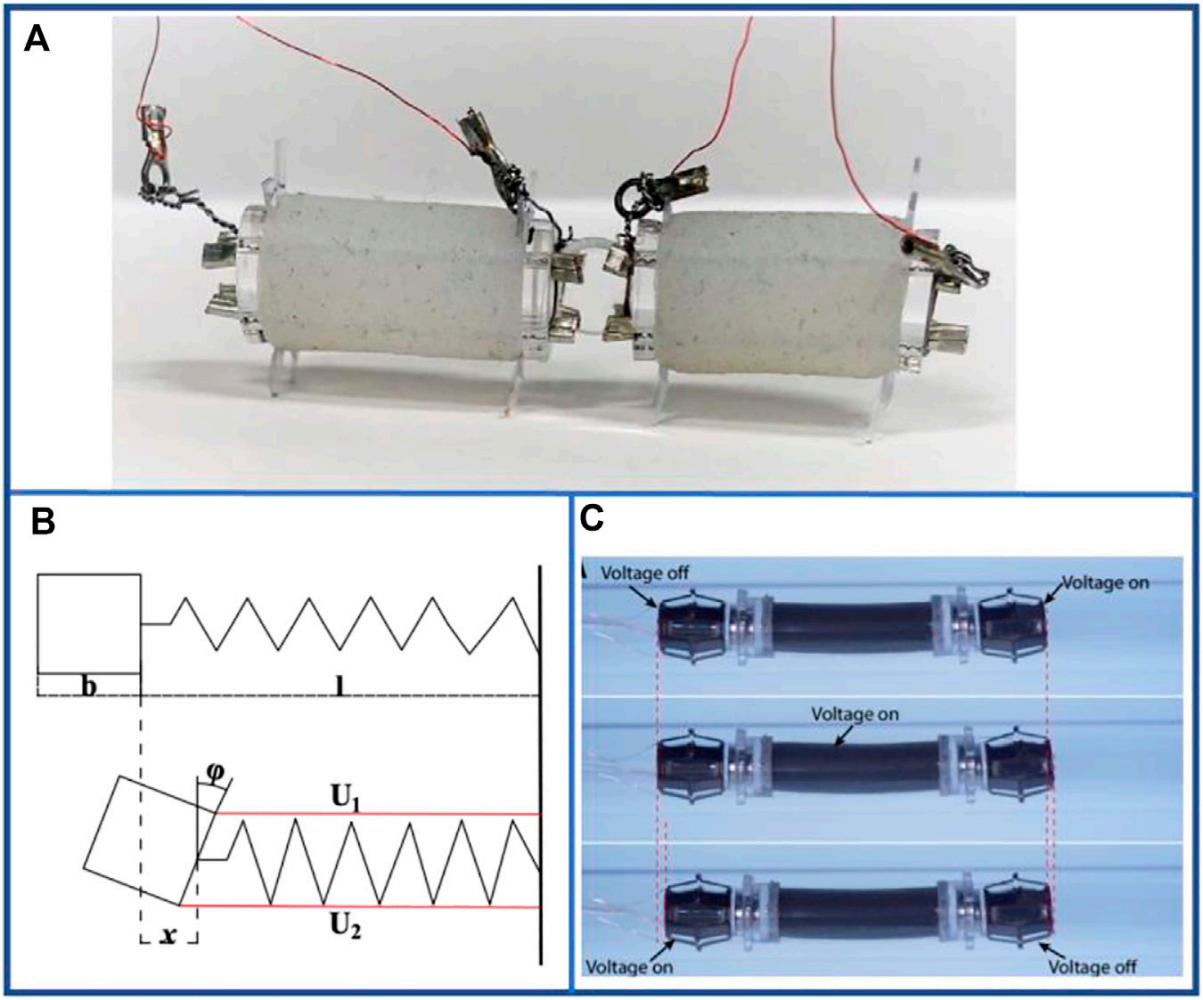
Examples of electro-deformable material-driven multi-segment earthworm-inspired soft robots. **(A)** Earthworm-like two-segment hydrostatic soft robot driven by SMA (Chatterjee et al., 2017). Reproduced from Ref. (Chatterjee et al., 2017). with permission. **(B)** The method of the rotary motion of robot with different applied voltages. **(C)** Structure and movement of sub-centimeter robot based on DE (Tang et al., 2022). Reproduced from Ref. (Tang et al., 2022). with permission.

This is because the characteristics of SMAs, i.e., elongation and contraction in response to temperature variation, they can be incorporated into deformable segments (Shinohara et al., 1999; Menciassi et al., 2004b; Liu et al., 2005; Saito et al., 2009; Chatterjee et al., 2017), just as shown in Figure 14A. For instance, preliminary tests have demonstrated that the earthworm prototypes designed by Menciassi et al. (2004b) have described a behavior similar to that of a biological earthworm and can attain a speed of 0.006875 bl·s^–1^. When the temperature drops, the SMA elongates with segmental axial elongation and longitudinal compression, and the temperature increases in case of an exactly opposite variation. This trend is extremely similar to the creeping of an actual earthworm and the parallel connection of the circuit eases timing control. Similarly, SMA-driven robots can achieve the steering function by initially adding two SMA segments into one deformable segment (Kheirikhah et al., 2012). Such a design can perform the telescoping function in a single direction under the same voltage loading, and it can twist to steer the robot when varying voltages are applied on these two SMA components as shown in Figure 14B. As this approach combines the steering design and straight travel design, it enables a higher degree of robot integration.

In context, dielectric elastomers (DEs) driven by high voltage can generate large strains. DE actuators (DEAs) can transform electric energy into mechanical work. Generally, the strains of DEA range from 10% to 35%, with a maximum of 300%. The electric-driven robots based on dielectric elastomers (Jung et al., 2007) can provide back-and-forth translational motion with two rotational degrees of freedom, including the advantages of reduced size, rapid response, no cables, and appropriate integration. Robots using dielectric elastomers can achieve sub-centimeter dimensions and are capable of rapidly movement in pipelines bearing complex geometries, filling media, and materials. Currently, the DE-driven robot shown in Figure 14C designed by Tang et al. (Tang et al., 2022) can achieve a rapid horizontal and vertical motion (horizontal: 1.19 body lengths per second; vertical: 1.08 body lengths per second) at a length of only 47 mm. Additionally, the voltage applied on this robot deforms the dielectric elastomer, allowing it to advance through the vibration inside the tube. Furthermore, the DE actuator designed by Lu et al. (2020) facilitates the elongation or contraction of the single segment, creating variable friction by contacting or separating from the ground, which is similar to the crawling process of an actual earthworm. The robot (Lu et al., 2020) can achieve back-and-forth movement with a maximum velocity of 0.077 bl·s^–1^ and a maximum velocity/mass ratio of 86.25 mm/(min^−1^ g^−1^).

### 4.4 Magnetic-driven

The magnetic-driven robots can be divided into two distinct types of control methods. First, addition of magnetic materials or coils for constructing the robot and driving the corresponding deformation of the robot or generating deformation by altering the direction and magnitude of the external magnetic field (Saga and Nakamura, 2002a; Wang et al., 2008; You et al., 2021). Second, twist the magnetic poles inside the robot using a motor or utilize the magnetic effect of current to increase the distance between the segments to produce deformation based on the principle of heteropolar repulsion (Saga and Nakamura, 2002b; Chi and Yan, 2003; Shin et al., 2011; Song et al., 2016).

**FIGURE 15:**
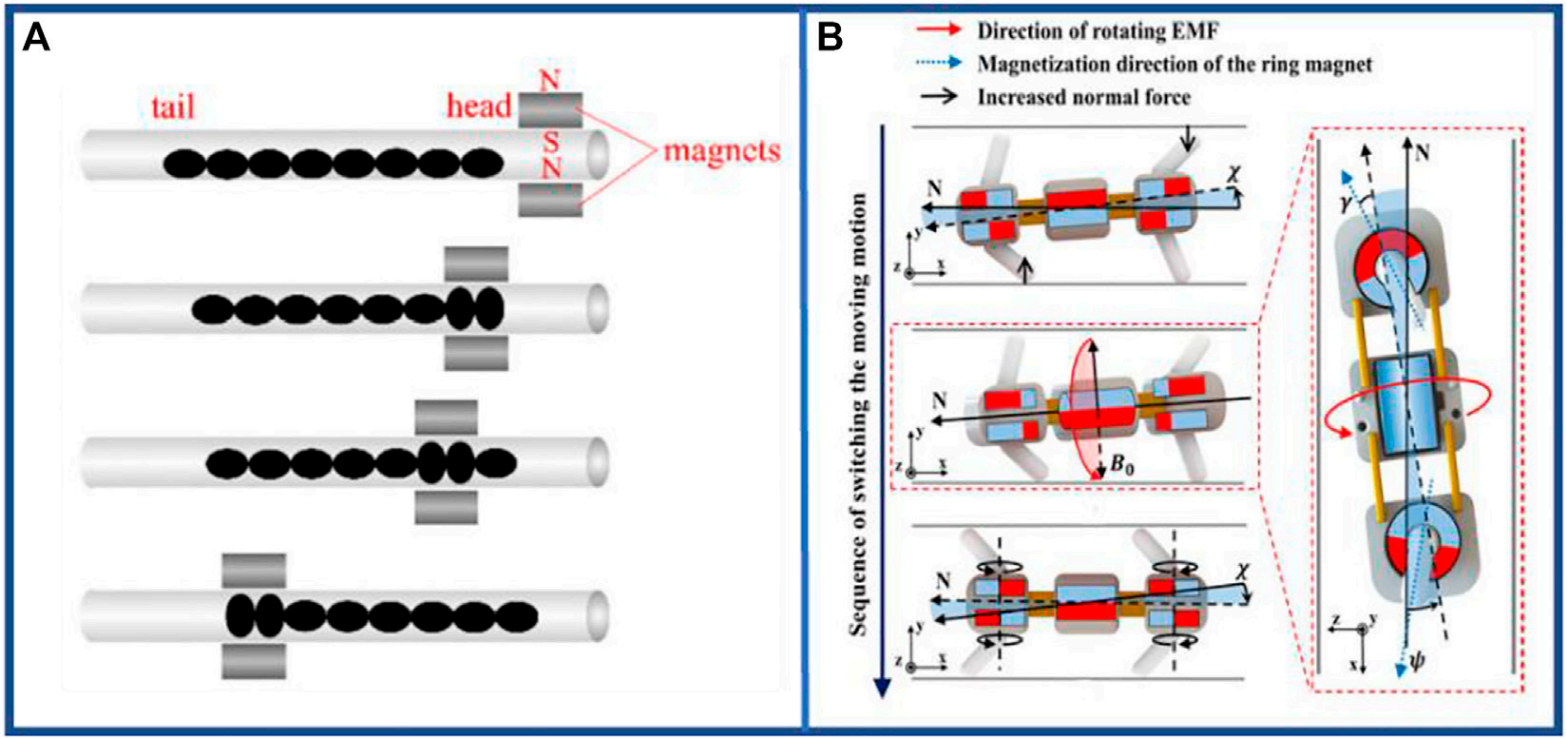
Examples of magnetic-driven multi-segment earthworm-inspired soft robot. **(A)** Mechanism of peristaltic locomotion with magnetic poles executing motion (You et al., 2021). Reproduced from Ref. (You et al., 2021). with permission. **(B)** Sequence to reverse motion direction by rotating the magnets (Jang et al., 2017). Reproduced from Ref. (Jang et al., 2017). with permission.

You et al. (2021) designed a 256 mm long robot that exhibits the highest crawler velocities for 0, 2, and 4 mm magnet separation—0.043, 0.05, and 0.0625 bl·s^–1^, respectively—for a magnet velocity of 1,250 mm s^–1^, just as shown in Figure 15A. In conclusion, these magnetic-driven robots, driven by an applied magnetic field, were generally light-weight and exhibited rapid response, enabling movement in complex environments.

The robots with interior magnets fundamentally followed the principle of “like poles repel and unlike magnet attract each other” to alter the distance between the segments and complete axial elongation. In addition, it shortened the distance between various segments and complete axial compression through dissimilar poles (Saga and Nakamura, 2002b; Chi and Yan, 2003; Shin et al., 2011). On a flat surface with a roughness of 0.75, the robot designed by Shin et al. (Shin et al., 2011) achieved an average speed of 1.31875 bl·s^–1^. To improve this performance, most researchers use a coil-magnet structure and switch the magnetism generated at one end of the coil by adjusting the current direction (Song et al., 2016).

Certain magnetic-driven robots have been designed based novel concepts. In earthworm bristle mimicry, twisting the magnetic poles can drive the rotation of bristle-like parts using a motor inside the segments (shown in Figure 15B) (Jang et al., 2017), or the applied magnetic field (Jung et al., 2021) can facilitate subsequent directional crawling. More importantly, the crawling motion of earthworms can be simulated by a deployable tensegrity structure (Yuan et al., 2021) such that the robot can smoothly travel in a smaller working area, whereas the folded size of this robot is small and the unfolded size is large. Furthermore, the unfolding and motion deformation of the robot can be driven by the magnetic force generated by its active member as an electromagnetic coil.

### 4.5 Hybrid-driven

Based on the advantages of the aforementioned actuation methods, a combination of these actuation methods can be practically used to form a hybrid-driven method. Owing to the divergent research maturity of pneumatic-, hydraulic-, electric-, and magnetic-driven method, a combination of pneumatic-driven and electric-driven is the main stream currently, whereas the other combinations still require further exploration.

For pipeline inspection, the pneumatic-driven parts allow the inflatable actuator to accommodate various pipe diameters, whereas the electric-driven actuators can significantly increase their operating speed (Consumi et al., 2022) to enable operation in occluded environments. For surface crawling, pneumatic actuators facilitate telescopic movement of the robot segment, whereas the electric-driven SMA can be programmed to control the telescopic motion of various SMAs on the same segment to achieve straight-line crawling or steering crawling (Zhang et al., 2021c).

**FIGURE 16:**
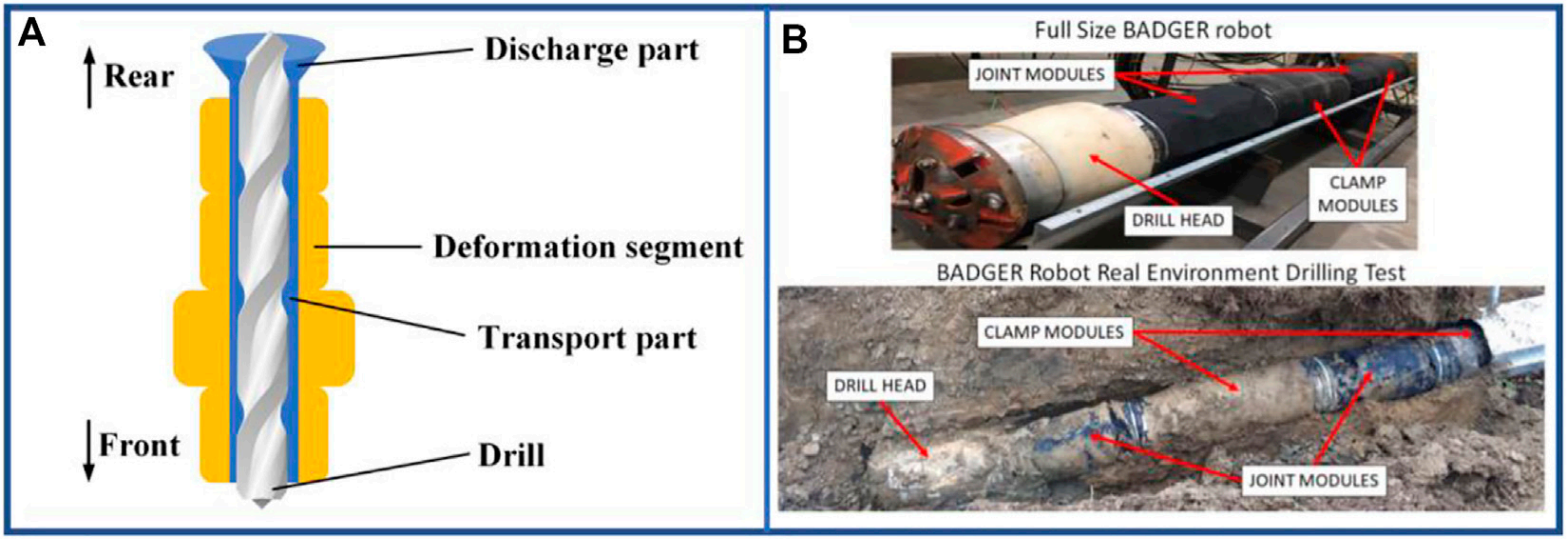
Examples of hybrid-driven multi-segment earthworm-inspired soft robot. **(A)** Diagram of underground explorer with drill and pneumatic actuator. **(B)** Robot BADGER hardware and its drilling test (Vartholomeos et al., 2021). Reproduced from Ref. (Vartholomeos et al., 2021). with permission.

Currently, the most prominent application scenario for hybrid-driven robots is soil drilling (Dewapura et al., 2020) (Omori et al., 2010; Omori et al., 2013; Nakatake et al., 2016; Isaka et al., 2019; Tokoi et al., 2021; Vartholomeos et al., 2021), and the basic structure is basically similar to that depicted in Figure 16A. The electrically controlled drill can dig forward, whereas the pneumatic actuator can compact the soil and form a certain diameter pipe channel, in addition to transporting the entire robot forward. This hybrid-driven robot shown in Figure 16B designed by Vartholomeos et al. (2021) is 3,200 mm long, and now is ready for industrial applications and can attain a speed of approximately 0.001875 bl·s^–1^.

### 4.6 Emerging actuation methods

With innovations in materials technology and the introduction of additional energy-conversion forms, refreshing types of actuation methods are now emerging in the field of earthworm-inspired robots.

**FIGURE 17:**
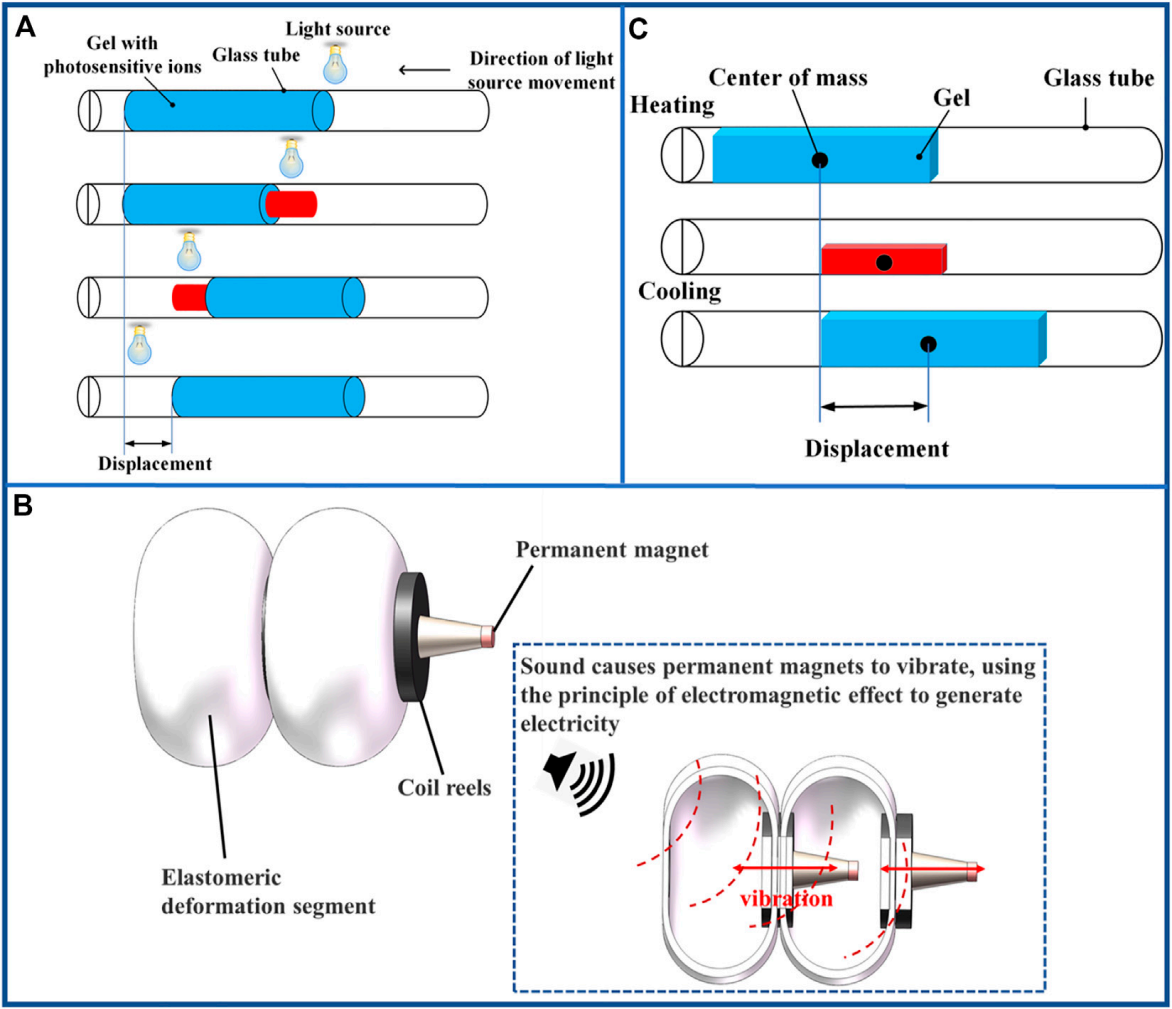
Examples of multi-segment earthworm-inspired soft robots using emerging actuation methods. **(A)** Motion of optical-driven earthworm-inspired robot. **(B)** Connection diagram and principle of common sound-driven earthworm-inspired robot. **(C)** Motion process and structure of thermal-driven robot.

In principle, the optical-driven, as the name implies, uses materials that can be deformed by light to accomplish expansion and contraction. Various photosensitive ions are embedded in the material and controlled by light (Sun et al., 2018), as shown in Figure 17A. The researchers could determine that cylindrically processed TiNS/AuNP hydrogels can mimic the movement of earthworms. A gel cylinder of 15 mm length achieved a displacement of 7 mm by a single laser scan. This actuation method offers the advantages of rapidity, large locomotion scale, and adequate repeatability, including the potential for long-distance manipulation in future.

Sound-driven places the actuating components in the actuators and vibrates the acoustic membrane through the transmission of sound (Nemitz et al., 2016), which consequently vibrates the coil for electromagnetic induction to generate electricity that drives deformation of the robot as shown in Figure 17B. This actuation method results in an earthworm-like writhing motion of the robot, which allows long-range manipulation and positioning. Moreover, it is highly beneficial in rescue scenarios.

Thermal-driven is usually used to produce deformation by changing the length as well as the thickness of the material through temperature changes, and the basic principle is the same as depicted in Figure 17C (Arora et al., 2009). Hydrogels, because of their ease of preparation and large material deformation at high temperatures, are often used to make thermal-driven earthworm-inspired robots. This actuation method can also make the heat induced deformed material deform without contact by means of heat conduction. However, the time required for cooling recovery of this robot is much longer than the time required for heating, so its deformation efficiency is relatively low.

## 5 Comparison of various actuation methods and locomotion forms

Based on the elaboration of the various actuation methods, their merits and demerits can be summarized as follows.

1. For pneumatic-driven, the earthworm-like motion can be accomplished through the self-extension of the soft material, and the steering motion can be accomplished by the air-chamber separation or Origami structure. This is a mature actuation method that offers adequate stability, safety, and environmental friendliness, which can be applied to both biotic and abiotic environments. However, existing pneumatic-driven mobile robots are tethered at all instances, because lightweight, small pneumatic pumps and valves are required to be developed. In addition, the compressibility of air, the non-linearity of fluid driven method, and the unpredictability deformation of hyperelastic material form the major constrains limiting the accuracy and mobility of the pneumatic-driven method.
2. For hydraulic-driven, it has the advantage of high output force and is the most similar method to actual earthworm, as it involves a liquid working medium. Specifically, it is highly suitable for high-pressure environments such as in deep ocean, because this method can easily balance the environmental pressure. Similar to pneumatic-driven, hydraulic-driven shares the disadvantage of non-linearity, which increases its control difficulty and limits its accuracy. Moreover, it poses an evident pollution problem of leakage.
3. In comparison, electric-driven can achieve precise control with low difficulty in related control and material affordability. More importantly, it can achieve continuous and consistent movements similar to the actual earthworm through certain continuous deformable structures. In contrast, this actuation method produces relatively higher overall hardness and its application in certain scenarios may be constrained, such as in human body. Owing to the extensive number of subdivision types, the advantages and disadvantages of the various subdivisions of electric-driven methods are discussed in Table 1.
4. For magnetic-driven, the material can be deformed and driven in a non-contact manner, or the body segments can elongate or contract by reversing the magnetic poles inside the segment. This actuation method exhibits a relatively high speed of movement and offers adequate safety in clinical use. However, this control method is inefficient and produces low output force.
5. For hybrid-driven, the most common form is the combination of pneumatic-driven and electric-driven, which bears the advantages of the above two actuation methods: high output force, rapid response, and fast movement. However, hybrid-driven produces control problems, and the overall size of the robot is particularly large. In addition to these issues, it is also difficult to complete the continuous and consistent change similar to the actual earthworm.
6. For the three emerging actuation methods, optical-driven has the advantage of rapid response and can achieve a similar peristalsis motion with the actual earthworm. However, the motion efficiency is relatively low and it offers limited application in closed environments such as pipelines. While the sound-driven can achieve remote control and positioning, it can be miniaturized with no built-in power module. However, this actuation method is still in the early stage of research, and the current material cannot be conveniently assembled. The thermal-driven can produce large deformation in a short time, but its cooling recovery time is too long, which makes the overall movement less efficient instead.

**TABLE 1:** Comparison between various types of actuation methods in earthworm-inspired soft robots.

Actuation methods	Subdivision actuation methods	Advantages	Limitations/Challenges
Pneumatic-driven	—	Soft, flexible, and continuous change	Unsuitable for high-pressure environment
		Fast, cheap, steerable, and controllable	Air supply line required
		Suitable for medical applications	Non-linearity and low accuracy
Hydraulic-driven	—	High stability	Non-linearity
		Suitable for high-pressure environment	Liquid input line required
		High output force	Pollution and leakage problems
Electric-driven	Direct motor-driven	Fast, steerable, and untethered	High hardness
		Precise control, and rapid response	Non-continuous motion
		High energy-conversion rate	Poor simulation of earthworm locomotion

Electric-driven	Wire-driven	Fast, steerable, and rapid response	Large volume occupation
		Linear control	High hardness
		Stable transmission and structure	
	Other continuous deformable structures	Appropriate simulation of earthworm locomotion	Large size
			Inferior structural stability
		Fast and large deformation	High construction difficulty
			Only suitable for surface locomotion
	SMA-driven	Flexible and precise control	Low efficiency
		Adequate stretchability	Sensitive to external temperature
	DE-driven	Fast movement speed	High degrees of hardness
		Small size, precise control and rapid response	High operating voltage
Magnetic-driven	—	Flexible, rapid response and continuous variations	Low efficiency
		Contactless control and large deformation	High material fabrication and control difficulty
		Convenient reverse movement	Low output force
Hybrid-driven	—	Fast, stable, and rapid response	Large volume occupation
		High output force	High control difficulty
		Suitable for industrial applications	High hardness
			Poor simulation of earthworm locomotion
Emerging actuation methods	Optical-driven	Soft, flexible, and untethered	Expensive and low efficiency
		Appropriate simulation of earthworm locomotion	Hard to fabricate
	Sound-driven	Soft, flexible, and untethered	Low efficiency
		Remote and contactless control	Hard to fabricate
		Large deformation	
	Thermal-driven	Soft, flexible, and untethered	Low efficiency
		Remote and contactless control	
		Large deformation	

In this Review, the various actuation methods and specific subcategories used for earthworm-inspired soft robots and the corresponding advantages and disadvantages are summarized in Table 1.

In addition to the aforementioned presentation, further forms of single- and multi-segment earthworm-inspired soft robots are summarized in Table 2. The aforementioned advantages, disadvantages, and structural innovations as well as their typical speeds, loading ability, and additional factors of performance will be discussed in the subsequent sections.

**TABLE 2:** Comparison between various locomotion forms used for earthworm-inspired soft robots.

Locomotion forms	Advantages	Limitations/Challenges
Single-segment	Simple structure, easy control, and low cost	Simple function
	High compactness and efficiency	Inferior stability and low efficiency
	Small-size and low power consumption	Limit of single behavior mode
		Non-continuous locomotion
Multi-segment	Diverse structures and interchangeability	High control difficulty
	Adequate fault tolerance	Relatively low movement speed
	Variable motion forms and functions	Manufacturing difficulty
	Outstanding carrying capacity and environmental adaptability	

## 6 Conclusion

**FIGURE 18:**
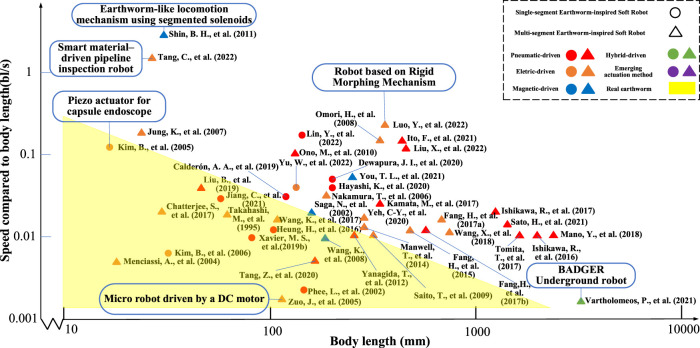
Comparison of standardized speed divided by body length of earthworm-inspired soft robots.

For earthworm-inspired soft robots, the speed of movement is an essential criterion for evaluating the locomotion ability. In the cited references, the speed metrics are standardized to speed divided by body length (bl/s) and plotted, as depicted in Figure 18. For hydraulic-driven soft robots, no detailed data is available for analysis, as they are predominantly in the theoretical research and experimental demonstration stage. Certain references do not provide specific motion speed values or the undeformed body length, owing to which, the speed after standardization cannot be displayed in this figure.

As portrayed in Figure 18, it can be concluded that most robots exhibit the standardized motion speeds ranging from 0.01 to 0.1 bl/s. Nearly half of the robots move at similar speeds to the actual earthworms (yellow triangle in Figure 18). Moreover, the motion speed of robots used in abiotic environments is typically faster than that of the robots used in biotic environments (Phee et al., 2002; Menciassi et al., 2004b; Zuo et al., 2005). The total length of robots used in the laboratory is generally between 10 and 1,000 mm, while the length of robots currently available for practical use is over 1,000 mm. Certain robots (Shin et al., 2011; Luo et al., 2022; Tang et al., 2022) have demonstrated remarkable locomotion ability that was much faster than the actual earthworm, which are the top-three robots in terms of standardized speed divided by body length. In Ref. (Tang et al., 2022), the dielectric elastomers were used for actuation and an adjustable periodic-high voltage was applied to control the material deformation for generating high-frequency vibrations in the pipeline. Ref. (Shin et al., 2011). proposed an actuator with a structure composed of cores and coils, and they used the magnetism generated by a threaded electric coil for actuation, thereby enabling high-speed locomotion and appropriate positioning performance, regardless of friction conditions. In contrast, Ref. (Luo et al., 2022). uses a linkage structure to produce large deformation driven by motor. In terms of quantity, traditional actuation methods, i.e., pneumatic- and electric-driven, have the largest number of references and are still the fundamental research direction. Overall, magnetic- and hybrid-driven exhibit immense potential, with appropriate speed characteristics and promising prospects for the future. In addition, three emerging driving methods rely on new discoveries in the field of novel materials and energy conversion methods that should be further explored in depth.

**FIGURE 19:**
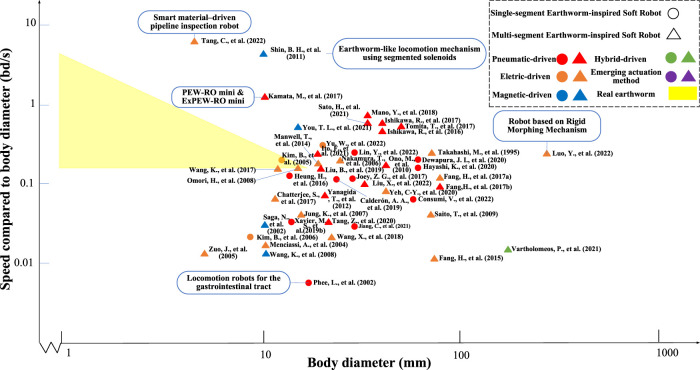
Comparison of standardized speed divided by body diameter of earthworm-inspired soft robots.

One of the main application areas of earthworm-inspired soft robots is pipeline inspection (medical endoscopy can also be considered for this purpose). Therefore, in order to compare the crawling performance of these robots inside the pipeline, a comparative analysis is also performed by the ratio of speed divided by robot body diameter (bd/s), as shown in Figure 19. This metric highlights the ability of the earthworm-inspired soft robot to pass through the pipeline. Similar to the data acquisition in the previous figure, some of the references do not explicitly give specific period of motion or the purpose of the robot is not for pipeline inspection. Therefore, the data after normalization for these robots is not be shown in this figure.

As portrayed in Figure 19, there is little correlation between the movement speed of the robot and that of actual earthworm. This is mainly because the actuation method and structure design of sub-centimeter level are still difficult to achieve. Most robots exhibit the standardized motion speeds divided by body diameter ranging from 0.01 to 1 bd/s, while their body diameter is mostly between 10 and 100 mm. Likewise, the motion speed of robots used in abiotic environments is typically faster than that of the robots used in biotic environments (Phee et al., 2002; Menciassi et al., 2004b; Zuo et al., 2005; Wang et al., 2008). DE-driven robot (Tang et al., 2022) and magnetic-driven robot (Shin et al., 2011) have no deformation in the diameter direction and can operate at high speed due to their high frequency vibration and rapid axial displacement respectively in pipeline. This indicates that robots used for pipeline inspection can move inside the pipeline by their own twisting/vibration or axial displacement, in addition to material expansion for movement. Moving by twisting/vibration lacks the step of material deformation, and its motion frequency as well as its own absolute speed are much higher. Other than that, pneumatic-driven robot (Kamata et al., 2017) illustrates that this traditional actuation method can still achieve high speeds despite with small diameters and that there is still room for continued innovation. It can be derived from the figure that the multi-segment robots have higher index, especially in the high-speed part, than the single-segment type in general. Although this conclusion differs from that shown in Figure 18, even though the absolute speed of the multi-segment robots is slower, it shows their better ability to pass through the pipeline. At present, there are few designs of large-diameter earthworm-inspired soft robots (Vartholomeos et al., 2021; Luo et al., 2022), which deserve further investigation and research. How to improve the ability of the soft robot to pass through the pipeline is also the key research direction in the future.

In Ref (Kandhari et al., 2021), in order to predict which control waveforms will achieve the highest rate of motion, cost of transport (COT) was proposed and can be used to characterize the body speed as a function of actuation rate of a single segment and waveform properties (number of waves). The optimal speed and the optimal waveform properties can be derived from the computational analysis performed by this function. Future earthworm-inspired soft robots can be optimized in terms of waveform drive (control method) and segment structure with a view to making the soft robot smaller and more responsive and achieving better motion efficiency.

In addition, loading capacity of earthworm-inspired soft robot is a crucial indicator. However, a standardized loading capacity calculation cannot be easily evaluated, because the lack of specific description of loading capacity in the references, or lack of their own self-weight data. However, certain references provide its specific load-bearing capacity. Its load-bearing capacity ranges from 1.47 N (Hu and Cheneler, 2021) to 52 N (Omori et al., 2013), whereas the robots themselves vary considerably in self-weight and actuation methods, thereby hindering the comparison based on a unified index.

Through the whole review, it can be found that even in the same actuation method, different structures can bring about different speed characteristics. Initially, the deformation is produced by the material itself (Qi et al., 2015), and the process of material expansion is exactly similar to the anchoring motion of the earthworm. At this point, anchoring and deformation of the robot is carried out through only one structure. Then a three-section deformable structure (SPID) emerges (Manfredi, 2021), which is the “anchoring-deformation-anchoring” structure. The key features of this structure is that the integral deformation is separated and can be targeted to optimize the telescopic part or the anchoring part. What’s more, the expansion or anchoring parts can be optimized accordingly. Optimizing the telescopic part, such as involvement of springs (Ito et al., 2021), bellows (Ono and Kato, 2010), etc., can increase the deformation of single segment, movement distance and speed. Similarly, by optimizing the anchoring part, the adaptability to different diameters can be increased and such optimization is an essential step for surface crawling robot (Joey et al., 2017). Subsequent structural innovations are no longer limited to common forms of the actual earthworm. The high-frequency vibration (Tang et al., 2022) or origami structure (Fang et al., 2017a), etc., have led to significant optimization of the structure and an enormous enhance in motion performance. With the introduction of additional manufacturing methods, finer structures were developed and used (Zhang et al., 2021b). At this point, the development of the robot focused mainly on the speed and efficiency of movement or COT. In recent years, structures such as linkage mechanisms (Horchler et al., 2015; Wang et al., 2018b; Kandhari et al., 2021) can be used to mimic the movement of the earthworm with good efficiency through one structure. The motion gait of robots with such structure is very similar to that of the actual earthworm, both having continuous and consistent features. It is worth noting that these structural optimizations do not indicate the superiority or inferiority. They all have the potential for further development and deserve further study.

Control methods and sensing innovations are also an important phase of robot development, but they are not mentioned because they do not correspond to the main topic of this review.

## 7 Future perspectives

The future development trend is analyzed according to different actuation methods. Relatively speaking, for non-complex actuation methods, pneumatic- and electric-driven earthworm-inspired soft robots are more maturely developed and suitable for structural improvement by these two traditional actuation methods. These two are also the most suitable and preferred actuation methods for researchers just involved in earthworm-inspired soft robotics, and they are also more efficient, safe and stable. In addition to this, the form of motion is also intuitively consistent with actual earthworm movement patterns. Other actuation method, like hydraulic-driven and magnetic-driven, for example, targeted R&D for specific environments and related expertise are required.

In future, pneumatic-driven robots can be optimized based on innovation in soft deformable structures to increase the displacement distance of a single cycle and output force, such as with the introduction of Origami and Kirigami structures (Fang et al., 2017b; Liu et al., 2019; Li et al., 2021). Simultaneously, the realization of untethered pneumatic-driven robots is a major issue that must be solved before they can be generally applied. This relies on the design of small pneumatic valves and air sources. Similar to hydraulic-driven methods that is a fluid driven method, the stated problems are common to pneumatic-driven methods as well. In particular, hydraulic-driven involves certain unique issues that need to be elaborated, such as leakage, pollution, and development of soft materials resistant to high pressure.

For generalized motor-driven robots, the overall hardness should be reduced to increase their similarity to real earthworms. The innovation of continuous deformable structures driven by motors is an appropriate solution. For electro-deformable material-driven robots, the materials with high deformability, insensitivity to ambient temperature, and low driving voltage can be developed or selected for actuation to achieve higher efficiency and improved safety.

For magnetic-driven robots, researchers can attempt to increase the controllability by modifying the magnetic material or adding a substance with large magnetic energy product such as Neodymium magnet. In addition, the structural design should be improved to reduce fabrication and control difficulties.

Currently, hybrid-driven robots are primarily formed by the combination of pneumatic- and electric-driven methods. Therefore, various other combinations of actuation methods are promising in future research. To improve the existing hybrid-driven robots, their overall hardness and volume occupation should be reduced.

Optical-driven method requires future optimization of materials to increase their operating efficiency and reduce the construction costs with the introduction of various photosensitive ions. Furthermore, sound-driven method can be developed to increase the operating speed by optimizing the electromagnetic conversion efficiency. While thermal-driven is inefficient, it is necessary to improve the deformation of materials at relatively high temperatures.

Interestingly, current trends have hinted at the combination of earthworm locomotion with other animal features (Du et al., 2022) to realize multibiological simulation. Combined with other driving units (e.g., propellers) (Fang et al., 2021), earthworm-inspired robots can archive multimodal motion, which considerably expands brand new usage scenarios.

In terms of application, both electric- and pneumatic-driven soft robots can be applied for pipeline inspection, medical endoscopy, and surface crawling purposes. However, in the medical field, magnetic-driven robots form an excellent choice, because these robots can be driven inside the biological gastrointestinal tract by an external magnetic field. This type of actuation method without a built-in power source offers a high degree of safety and can be fabricated at an extremely small size, which is in line with the current requirements of the medical field. Thus, hydraulic- and hybrid-driven robots are suitable for pipeline inspection or soil drilling, because they can fulfill the high-output force requirements or work in high-pressure environments. Optical-driven is suitable for pipeline inspection and surface crawling under light condition. Non-etheless, sound- and thermal-driven robots are more likely to be used for remote control, as they can be driven without any contacting.

For future structural optimization, motion efficiency needs to be put in the first place. For example, not only can the number of control or input components be reduced through structural optimization, but also can new structures be considered to make the robot move longer distance in single cycle. Anchoring segments need to take advantage of not only radial expansion but also anisotropic friction or a changing friction coefficient especially for surface crawling. Optimization for the miniaturization of valves and control elements is equally important. Making soft robots more autonomous, more sensitive, and ultimately more like actual earthworms is the ultimate goal in the development of such robots.

In conclusion, for earthworm-inspired soft robots, there is still room for improvement in the future. As for the improvement of the actuation method, pneumatic valve miniaturization, the difficulty of material preparation and other issues are still urgent problems to be solved. In a normalized comparison of the motion performance of the robots in this review, there is still a gap between mainstream robots developed so far and actual earthworms, especially in the motion speed divided by body diameters. Thus, the performance improvement still needs to be continued.
